# Coexisting Molecular Determinants of Acquired Oxaliplatin Resistance in Human Colorectal and Ovarian Cancer Cell Lines

**DOI:** 10.3390/ijms20153619

**Published:** 2019-07-24

**Authors:** Paul Noordhuis, Adrianus C. Laan, Kasper van de Born, Richard J. Honeywell, Godefridus J. Peters

**Affiliations:** Department of ^1^Medical Oncology, Amsterdam UMC, Location VU University Medical Center (VUmc), CCA 1.52, De Boelelaan 1117, 1081 HV Amsterdam, The Netherlands

**Keywords:** oxaliplatin resistance, platinum accumulation, platinum DNA adducts, gene expression, array comparative genomic hybridization

## Abstract

Oxaliplatin (OHP) treatment of colorectal cancer (CRC) frequently leads to resistance. OHP resistance was induced in CRC cell lines LoVo-92 and LoVo-Li and a platinum-sensitive ovarian cancer cell line, A2780, and related to cellular platinum accumulation, platinum-DNA adducts, transporter expression, DNA repair genes, gene expression arrays, and array-CGH profiling. Pulse (4 h, 4OHP) and continuous exposure (72 h, cOHP) resulted in 4.0 to 7.9-fold and 5.0 to 11.8-fold drug resistance, respectively. Cellular oxaliplatin accumulation and DNA-adduct formation were decreased and related to OCT1-3 and ATP7A expression. Gene expression profiling and pathway analysis showed significantly altered p53 signaling, xenobiotic metabolism, role of BRCA1 in DNA damage response, and aryl hydrocarbon receptor signaling pathways, were related to decreased ALDH1L2, Bax, and BBC3 (PUMA) and increased aldo-keto reductases C1 and C3. The array-CGH profiles showed focal aberrations. In conclusion, OHP resistance was correlated with total platinum accumulation and OCT1-3 expression, decreased proapoptotic, and increased anti-apoptosis and homologous repair genes.

## 1. Introduction

Oxaliplatin is a third generation analogue of cisplatin and has shown remarkable clinical activity in colon cancer with intrinsic resistance to cisplatin. Oxaliplatin is used in combination with other drugs including 5-fluorouracil with leucovorin (FOLFOX), resulting in response rates of up to ~60% [1] and for pancreatic cancer the addition of irinotecan (FOLFIRINOX) led to an improved therapy [2]. Platinum-DNA adducts mediate the pharmacological activity of oxaliplatin, leading to growth arrest and subsequent apoptosis [3,4,5]. Similar to cisplatin, oxaliplatin resistance is achieved via several modalities including reduced drug uptake and/or enhanced efflux of the drug, intracellular sequestration, decreased DNA adduct formation, increased DNA repair, or increased adduct tolerance and reduced response to the platinum DNA adducts [6,7,8,9]. Cellular accumulation of oxaliplatin is a net result of uptake and efflux. Oxaliplatin uptake can be mediated by the human copper transporter hCTR1, as well as by the organic cation transporters OCT1, 2, and 3 (SLC22A1-3) [10,11,12]. For the efflux or sequestration of oxaliplatin, P-type ATPases including ATP7A and ATP7B appear to play a functional role [10,13,14]. As a consequence of the decreased oxaliplatin transport, the formation of platinum-DNA adducts may be diminished. Differences in platinum DNA adducts and downstream signaling possibly explain the activity in colon cancers that are intrinsically resistant to cisplatin [5,15,16]. For full pharmacological activity, cisplatin relies on the mismatch repair (MMR) system. In contrast, oxaliplatin is also active in MMR-deficient cells [15,17,18,19]. The nucleotide excision repair system (NER) involved in repair of oxaliplatin-DNA adducts has been shown to have a predictive value for the treatment of colorectal cancer [9,20,21]. Downstream factors that influenced sensitivity or resistance to oxaliplatin include defects in proapoptotic genes Bax or delayed activation of both Bax and Bad, and subsequent reduction of mitochondrial transmembrane potential [5,16,22,23]. The importance of the mitochondria to oxaliplatin activity was further demonstrated in enucleated cells where oxaliplatin retained its ability to induce apoptotic response indicating that there are multiple mechanisms of action [22]. In the apoptotic response, p53 plays an important role and it has been demonstrated that inactivation or mutated p53 alters the cytotoxicity of cisplatin [24,25,26]. Although targeted inactivation of p53 in HCT116 cells decreased the activity of oxaliplatin, it was not possible to predict sensitivity based on p53 status in a group of 30 colorectal cancer cell lines [27].

Clearly, subtle differences in determinants of cisplatin and oxaliplatin resistance exist which may be tissue specific and possibly influenced by p53 expression. To further dissect out mechanisms of oxaliplatin resistance we induced resistance in colon cancer cells with different p53 functional status and compared this to ovarian cancer cells. Resistance towards oxaliplatin was studied in relation to common mechanisms of drug resistance including cellular drug accumulation; expression status of relevant uptake and efflux transporters; and formation, retention, and repair of platinum DNA-adducts. Since the underlying pathways that mediate resistance towards oxaliplatin are poorly described, we also investigated alterations in gene expression in the drug-resistant cell lines and explored whether or not these changes are a result of aberrations at the genomic level.

## 2. Results

### 2.1. Growth Inhibition

**Figure 1:**
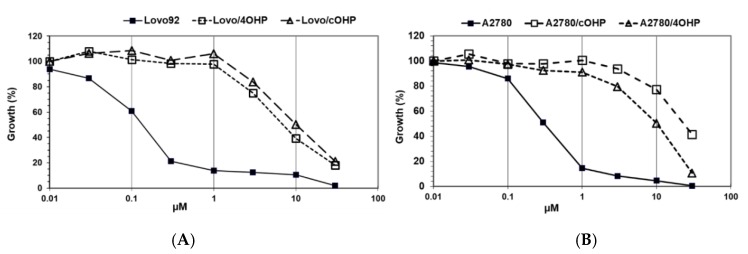
Sensitivity of Lovo-92 (**A**) and A2780 (**B**) parent cell lines and their oxaliplatin resistant variants 4OHP and cOHP. Curves are from a representative experiment performed in triplate out of at least three separate experiments.

The results of the growth inhibition experiments aimed at determining the degree of drug resistance induced by exposure to gradually increasing concentrations of oxaliplatin over seven months (Figure 1). Since our research question focused on the potential differences with cisplatin, we determined cross-resistance to cisplatin as shown in Table 1. However, we did not include other DNA damaging drugs, predominantly because they have different transport properties and because their DNA damage is repaired by different DNA repair enzymes. Interestingly, preliminary results showed an increased sensitivity to x-irradiation.

The colon cancer cell line, LoVo-92, was most sensitive to oxaliplatin with an IC_50_ value of 0.21 μM. Induction of resistance after four hours of oxaliplatin pulses resulted in a 7.9-fold resistance and after continuous drug exposure resulted in a 11.8-fold resistance. The LoVo-Li cells that express inactive p53 were 3.5-fold less sensitive to oxaliplatin and the resistant variants showed a similar pattern of resistance towards oxaliplatin as the wt p53 parental cell line LoVo-92 with resistance factors of 4.0 and 5.6 for LoVo-Li/4OHP and LoVo-Li/cOHP, respectively. Remarkably, these LoVo-92 and LoVo-Li variants retained parental cell sensitivity to cisplatin. Moreover, the oxaliplatin-resistant sublines of the ovarian cancer cell line A2780 displayed higher resistance levels after continuous exposure to oxaliplatin rather than after four hours of pulses, hence being 11.1- and 5.3-fold, respectively. In contrast to the colon cancer cell lines, these oxaliplatin-resistant ovarian cancer cells were also cross-resistant to cisplatin; 7.0- and 9.3-fold for A2780/4OHP and A2780/cOHP, respectively. However, resistance to l-OHP was lower than that of CDDP in a CDDP-resistant variant, ADDP, which was included for comparison. In order to determine the stability of the resistance, we also cultured the cells for several months without l-OHP and determined the sensitivity to l-OHP, but no loss of resistance was found. Although we did not clone our cells, this result indicates that induction of resistance led to a homogenous, resistant cell population.

### 2.2. Cellular Oxaliplatin Accumulation and Formation of DNA Adducts

**Figure 2:**
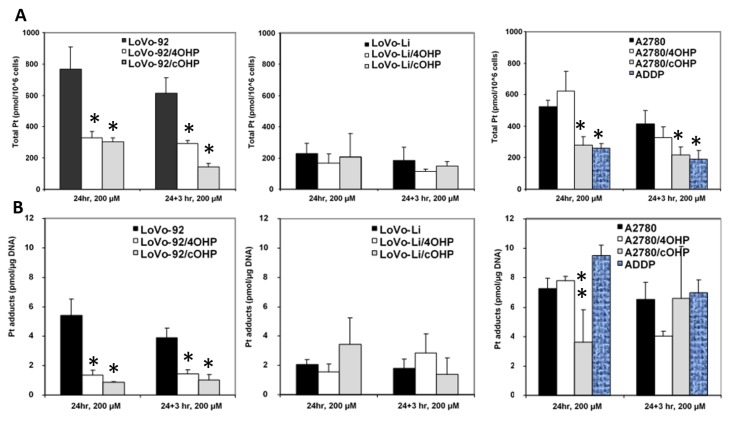
Total platinum accumulation (**A**) and platinum DNA adducts formation (**B**) in LoVo-92 variants, LoVo-Li variants, and A2780 variants. ADDP is a CDDP resistant variant of A2780. Values are means ± SEM of at least 3 experiments and expressed as pmol/10^6^ cells (**A**) and as pmol/μg DNA (**B**). Cells were exposed for 24 h to 200 μM oxaliplatin and, in parallel, for 24 h followed by 3 h in a drug-free medium. Significant changes (*p* < 0.05) compared to parental cell lines are marked with *, while ** was close to significance (*p* = 0.055).

In order to determine whether or not oxaliplatin resistance is mediated by reduced drug uptake or increased efflux, which might result in decreased formation of platinum DNA adducts, cellular drug accumulation and DNA adduct formation were determined (Figure 2). Earlier, we used both 20 and 200 μM l-OHP [28] to determine the accumulation of drug and formation of platinum adducts. Similar relative data were found at both concentrations. However, since the sensitive parent cells might not tolerate an exposure to a high 200 μM l-OHP, we also exposed the cells to 20 μM for 4 h. Indeed 24 h exposure to 200 μM resulted in 25% dead cells, but no dead cells were found after 4 h exposure to 20 μM l-OHP, even in the sensitive A2780 wild type. Unfortunately, this concentration was too low and the time too short to measure detectable Pt-adduct levels in the resistant cells with the FAAS method. However, in the sensitive wild type cells, Pt-adduct levels were detectable, albeit, much lower than that at 200 μM. For the retention experiments, Pt-adduct levels were too low to be detectable after 3 h. Recalculation showed that the Pt-adduct levels at 200 μM l-OHP levels were linearly related to the 4 h 20 μM l-OHP adduct levels. Therefore, all statistical evaluations were done with data obtained at 200 μM l-OHP. Levels in the resistant variants, LoVo-92/4OHP and LoVo-92/cOHP, showed a marked reduction (~60%) in cellular accumulation of platinum. We also explored the possibility of increased platinum efflux; hence, after removal of the drug and incubation for 3 h in drug-free medium, LoVo-92/4OHP showed a 10% reduction in the accumulated oxaliplatin, similar to parental LoVo-92 cells, whereas, in LoVo-92/cOHP a reduction of 50% was observed suggesting that increased efflux of oxaliplatin might contribute to resistance to oxaliplatin. The LoVo-Li cells, which were less sensitive as compared with LoVo-92, showed a 4-fold lower accumulation of oxaliplatin. However, no significant change in cellular accumulation of oxaliplatin was observed in the oxaliplatin-resistant variants. Removal of the drug and incubation for 3 h in a drug-free medium showed 30% reduction in both LoVo-Li/4OHP and LoVo-Li/cOHP as compared with 20% for the parental LoVo-Li cells. The ovarian cancer cell lines showed a mixed picture; there was no significant change in accumulation after 24 h exposure in A2780/4OHP cells, whereas, A2780/cOHP accumulation was about 50% as compared with the parental cell line A2780. Total platinum levels after removal of the drug showed a similar decrease for both parental A2780 and oxaliplatin resistant A2780/cOHP. The A2780/4OHP cells showed a 50% decrease of total platinum levels after incubation in a drug-free medium suggesting that increased oxaliplatin efflux may be a contributing factor to the drug-resistant phenotype.

Consistent with the decreased oxaliplatin accumulation, formation of platinum-DNA adducts was also decreased in LoVo-92/4OHP and LoVo-92/cOHP (Figure 2B). Removal of the drug and incubation in a drug-free medium did not show decreased DNA adducts, suggesting that increased repair does not contribute to resistance in these cell lines. Consistent with the accumulation of oxaliplatin in LoVo-Li/4OHP and LoVo-Li/cOHP cells, the formation of DNA adducts was also not significantly altered both after 24 h exposure as well as after additional incubation in a drug-free medium. The formation of platinum DNA adducts in A2780/4OHP and A2780/cOHP reflected the pattern of total platinum accumulation in these cell lines. A2780/4OHP showed somewhat higher content of DNA adducts which might be expected of the somewhat higher accumulation of the drug, whereas, in A2780/cOHP cells, formation of DNA adducts was reduced to an extent that was similar to that of cellular platinum accumulation.

**Figure 3:**
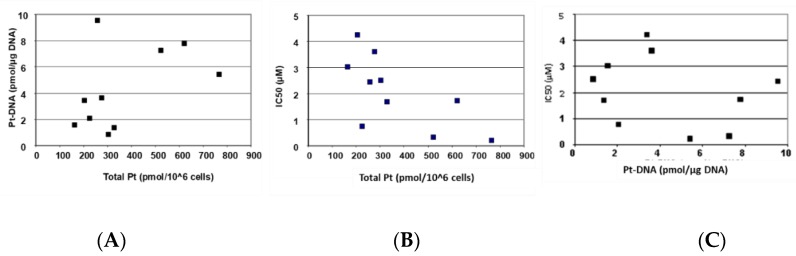
Relationship between total platinum accumulation, platinum DNA adducts formation, and sensitivity towards oxaliplatin in parental and oxaliplatin resistant cell lines. (**A**) Scatter plot of total platinum accumulation (pmol/10^6^ cells) and formation of platinum DNA adducts (pmol/μg DNA), (**B**) scatter plot of total platinum accumulation (pmol/10^6^ cells) and sensitivity (μM) towards oxaliplatin and (**C**) scatter plot of platinum DNA adducts formation (pmol/μg DNA) and sensitivity (μM) towards oxaliplatin. The IC50 values are taken from Table 1 and total platinum accumulation and platinum DNA adducts are from Figure 2.

Figure 3 depicts the correlation of cellular oxaliplatin accumulation, DNA adduct formation, and oxaliplatin sensitivity. Figure 3A and Table 2 show that an increased cellular accumulation of oxaliplatin resulted in a consistently and significantly higher formation of DNA adducts. Figure 3B,C and Table 2 show that sensitivity towards oxaliplatin is highly and significantly correlated with cellular accumulation of the drug but not with the formation of platinum DNA adducts.

### 2.3. Gene Expression of Relevant Transporters and DNA Repair Genes

**Figure 4:**
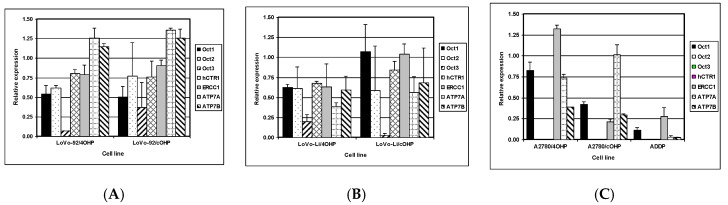
mRNA expression of transporters and DNA repair genes as determined by Q-PCR in (**A**) LoVo-92 variants, (**B**) LoVo-Li variants, and (**C**) A2780 variants. Values are means of two experiments in triplicate and are expressed relative to the house keeping gene β-actin.

The net cellular accumulation of oxaliplatin and consequent formation of platinum DNA adducts might be affected by influx and efflux transporters as well as DNA repair. Therefore, mRNA expression of the copper transporter, hCTR1; the organic cation transporters OCT1, 2, and 3 (SLC22A1-3); as well as the efflux transporters ATP7A and ATP7B were determined by Q-PCR. The expression of nucleotide excision repair gene ERCC1 was also evaluated (Figure 4). The results for LoVo-92 and the resistant variants reflect the results of cellular oxaliplatin accumulation and DNA adduct formation experiments. Changes in gene expression levels in LoVo-92/4OHP and LoVo-92/cOHP were almost similar and showed decreased expression of the influx transporters hCTR1 and OCT1, OCT2, and OCT3 with 20%, 50%, 30%, and 20%, respectively. ERCC1 gene expression was decreased by 15%, whereas, expression of the efflux transporters ATP7A and ATP7B was increased by 30% and 20%, respectively.

LoVo-Li cells displayed lower gene expression levels of influx transporters corresponding with lower drug accumulation and formation of oxaliplatin DNA adducts in LoVo-Li cells as compared to LoVo-92. The LoVo-Li/4OHP cells showed decreased expression of all influx and efflux transporters, as well as the DNA repair gene ERCC1. The hCTR1, OCT1, OCT2, and ERCC1 were decreased by 40%, whereas, OCT3 showed a decreased expression of 80%. ATP7A and ATP7B were decreased by 60% and 40%, respectively. The LoVo-Li/cOHP OCT1 and ERCC1 genes were unchanged, whereas, the other genes showed a decreased expression. Expression of the influx transporters hCTR, OCT2, and OCT3 were decreased by 15%, 40%, and 95%, respectively. The efflux transporters, ATP7A and ATP7B, showed a decreased expression of 45% and 30%, respectively. The drug-resistant ovarian cancer cell lines exhibited decreased expression of all transporter genes except for OCT2 and OCT3 which were not detectable in the parental cells, as well in the resistant cells. Expression of OCT1 in A2780/4OHP and A2780/cOHP was decreased by 20% and 60%, respectively.

ATP7A was decreased by 25% in A2780/4OHP but unchanged in A2780/cOHP. ATP7B was decreased by 35% in both A2780/4OHP and A2780/cOHP. The nucleotide excision repair gene ERCC1 showed an increased expression in A2780/4OHP but was decreased by 80% in A2780/cOHP. The Pearson correlation analysis (Table 2) of the Q-PCR data with total platinum accumulation, platinum DNA adducts formation, and sensitivity to oxaliplatin, showed that OCT1 was significantly correlated with total platinum accumulation for all cell lines, whereas, OCT2 and OCT3 were significantly correlated with total oxaliplatin accumulation in the LoVo cell lines. Remarkably, ERCC1 was positively and significantly correlated with the formation of platinum DNA adducts. In addition, using the Spearman correlation analysis, a significant correlation was found between the gene expression of the copper transporter hCTR1 and total platinum accumulation, whereas, ATP7A was significantly correlated with formation of platinum DNA adducts. Furthermore, the expression of OCT2 and OCT3 were significantly correlated with sensitivity to oxaliplatin.

### 2.4. Genome-Wide Expression Array Analysis

Although in LoVo-92 variants, total platinum accumulation, DNA adducts formation, and transporter gene expression appear to correlate and provide adequate explanation for drug resistance, however, the picture for LoVo-Li and A2780 variants is less clear. We, hence, performed gene expression arrays to gain better insight into the mechanisms underlying oxaliplatin resistance. The gene expression array data were significantly correlated with the quantitative gene expression data obtained.

The Q-PCR data for the genes are described above. Ingenuity pathway analysis (IPA) of normalized log2 ratios of the resistant cell lines and corresponding parental cell line (gene expression omnibus: GSE19992) was used to pinpoint the canonical pathways that were most significantly enriched. The most frequently and significantly enriched pathways are shown in Table 3. Gene expression levels of the most relevant pathways are shown in Supplemental Table S1.

#### 2.4.1. Axonal Guidance Signaling and Aryl Hydrocarbon Receptor Signaling

The axonal guidance signaling and aryl hydrocarbon receptor signaling pathways were significantly enriched for four out of six resistant cell lines. The expression of genes of the ALDH family was most frequently altered in the aryl hydrocarbon receptor signaling pathway. ALDH1L2 was decreased in all cell lines with log2 ratios from −2.26 for A2780/4OHP to −3.45 for LoVo-Li/4OHP. The ALDH1A2 gene was increased in all cell lines except for the LoVo-Li/cOHP. Unexpectedly, resistant ovarian cancer cell lines showed a decreased expression of the glutathione-S-transferase family of genes. However, microsomal glutathione-S-transferase 1 showed an increased expression in these resistant variants and to some extent also in drug-resistant colorectal cancer cell line variants.

#### 2.4.2. p53 Signaling

Various signaling pathways showed major changes in the expression of their pertaining genes, which may, in general, point to an altered, mostly reduced capacity to induce cell death, as summarized in Table 4. The p53 signaling pathway was significantly enriched in LoVo-Li/4OHP and in all the resistant ovarian cancer cell lines. A decreased expression of the proapoptotic gene, Bax, was the most common altered gene. Moreover, other proapoptotic genes, such as BBC3 (Puma) and Apaf1, also showed a decreased expression. PCNA, involved in replication and DNA damage response showed an increased expression both in resistant colorectal cancer cell lines as well as in ovarian cancer cell lines. A decreased expression of the tumor suppressor, SerpinB5, was only observed in resistant colorectal cancer variants. However, other genes involved in the p53 signaling pathway showed inconsistent changes. The proapoptotic gene, PMAIP1 (Noxa), was increased in A2780/4OHP. Cell death executors, Caspase 6 and 7, were increased in gene expression in the ovarian cancer cell lines and only modest in LoVo-Li/4OHP, but in LoVo-Li/4OHP this was accompanied by an increased expression of the inhibitor of the apoptosis (IAP) gene, BIRC5 (Survivin), and decreased expression of BIRC4 (XIAP). The ovarian cancer cell lines showed increased BIRC4 and BIRC5 expression in A2780/4OHP and A2780/cOHP, respectively. Other genes that were strongly increased in the ovarian cancer cell lines were HIPK2 and PLAGL1. p53 was only decreased in A2780/4OHP and A2780/cOHP. The p53 induced nuclear protein 1, TP53INP1, showed decreased gene expression in all cell lines but LoVo-92/4OHP. Taken together, the proapoptotic pathway seemed decreased and the anti-apoptotic pathway increased, thereby possibly resulting in an overall decreased apoptotic potential in the drug-resistant cell lines.

#### 2.4.3. Role of BCRA1 in DNA Damage Response

Less frequently observed yet highly significant for the drug-resistant variants of LoVo-Li is the role of BCRA1 in DNA damage response, metabolism of xenobiotics by cytochrome p450, xenobiotic metabolism signaling pathways, and G1/S checkpoint regulation. The role of BRCA1 in DNA damage response was most prominent in LoVo-92/cOHP and LoVo-Li/4OHP, but the ovarian cancer cell lines also showed altered expression of genes in this pathway. Excluding E2F5, all genes in this pathway were increased in LoVo-Li/4OHP, whereas, in LoVo-92/cOHP increased expression of BLM, CHEK1, E2F1, Rad51, and RFC2 was observed. In the ovarian cancer cell lines A2780/4OHP and A2780/cOHP the highest increase was observed for FANCA.

#### 2.4.4. Xenobiotic Metabolism Signaling Pathways and G1/S Checkpoint Regulation

Both xenobiotics metabolism pathways were only significantly enriched for LoVo-Li/4OHP but included genes that were found to be increased in other resistant variants as well. Most prominent was the above described ALDH family of genes. Other frequently increased genes were aldo-keto reductase family members 1C1 and 1C3 which were increased in four out of six and all drug-resistant variants, respectively. The expression of the multidrug efflux transporter, ABCC2 (MRP2), was increased in oxaliplatin-resistant ovarian cancer cell lines, however, decreased gene expression was observed in resistant LoVo-Li variants. However, resistant LoVo-Li variants showed increased expression of another MDR efflux transporter, ABCB1 (Pgp). The G1/S checkpoint regulation was only significant for LoVo-Li/4OHP and showed increased expression of cyclins D1 and D3; cyclin dependent kinases 2 and 4; E2F family members 1, 3, and 4 and also RBL1; and the transcription factor TFDP1.

### 2.5. Oxaliplatin Induces Chromosomal Aberrations

To determine whether alterations in gene expression could be the result of gene copy number variations, we performed aCGH. The chromosomal profiles (normalized log2 ratio) are shown in Supplemental Figure S1 including aberration calls of the resistant cell lines compared to parental cell lines, as well as profiles of parental cell lines compared to reference DNA (gene expression omnibus: GSE20144). Common or overlapping aberrations are summarized in Table 5.

Analysis of aCGH data for common aberrations revealed that eight out of 10 observed common or overlapping aberrations are found in drug-resistant cell lines derived from the same parental cell line. Focal gains observed in Chr 15 and Chr 16 showed that similar aberrations are also found in both resistant colon and ovarian cancer drug-resistant cell lines. Remarkably, parental LoVo-92 cells displayed a gain at Chr15 which was completely lost in LoVo-92/4OHP and partly lost in LoVo-92/cOHP cells.

## 3. Discussion

Oxaliplatin resistance is a major clinical hurdle for curative chemotherapy since it is a crucial component of combination regimens for colorectal cancer. Although much knowledge has been gained regarding mechanisms underlying resistance to the first-generation platinum compound, cisplatin, those relating to oxaliplatin resistance are still not well characterized.

Since the pharmacological activity or resistance to oxaliplatin might be affected by tissue type and treatment schedule as well as by other factors including p53 status, we established oxaliplatin-resistant cell lines representing these parameters. The colorectal cancer cell lines LoVo-92 and its variant LoVo-Li, as well as the ovarian cancer cell line, A2780, were made resistant via short-term drug pulses to reflect the clinical bolus administration and compared the latter to cell lines which acquired resistance after continuous exposure to oxaliplatin. The data show moderate levels of resistance (i.e., clinically relevant) ranging from four- to 12-fold as compared with parental cells. Remarkably, low level cross-resistance towards cisplatin was observed in the colorectal cell lines, whereas, oxaliplatin-resistant ovarian cancer cell lines showed similar resistance levels for both cisplatin and oxaliplatin. This observation may be explained by the intrinsic cisplatin resistance of both parental colorectal cancer cell lines which are deficient in mismatch repair and an established mechanism of resistance to cisplatin but not oxaliplatin [17,18,19,29]. Therefore, induction of resistance to oxaliplatin may not necessarily result in cross-resistance to cisplatin. Since cisplatin-resistance levels were similar to those of oxaliplatin in the oxaliplatin-resistant ovarian cancer cell lines, it is likely that similar resistance mechanisms may be operative that play a role for both platinum drugs. We may speculate that resistance to oxaliplatin in ovarian cancer may look like that in A2780 cells, and that oxaliplatin resistance in colorectal cancer may reflect that of LoVo and other colorectal cancer cells, described earlier [28].

**Figure 5:**
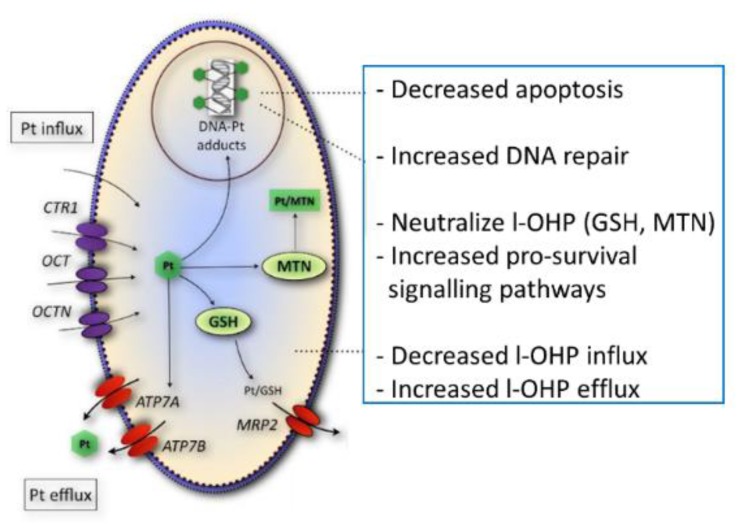
Potential resistance mechanisms for oxaliplatin (l-OHP). Decreased accumulation of l-OHP into the cell is related to a decreased uptake by one of the OCTs or CTR1, while the ATPases, ATP7A, and ATP7B, and MRP2 can efflux platinum (Pt) analogs, including l-OHP. The drug can also be neutralized by glutathione (GSH) or MTN (metallothionein protein). Alternative pathways of resistance may be mediated by a decreased expression of proapoptotic proteins and an increased prosurvival signaling, such as ALDH1 and Akt [16].

Although both cisplatin and oxaliplatin are believed to exert their cytotoxic activity via formation of DNA adducts, involvement of mismatch DNA repair is different for each drug, although for both cisplatin and l-OHP a decreased accumulation was found in resistant cells [30]. Moreover, other pharmacological parameters, such as drug transport, have been reported to be different for both platinum compounds [12,31]. Such studies have been performed either with cells with acquired resistance or by knocking out several important genes such as repair enzymes or transporters. These studies clearly highlighted the importance of these processes. On the basis of these studies we focused on the role of these parameters in oxaliplatin resistance. Figure 5 shows a summary of several potential resistance factors for oxaliplatin. Accumulation of total platinum was significantly correlated with sensitivity to oxaliplatin in this panel of parental- and drug-resistant cell lines in contrast to the formation of platinum DNA adducts, suggesting that alternative drug targets are important for oxaliplatin activity. Since gene expression levels of organic cation transporters were significantly correlated to total platinum accumulation, it is likely that the loss of the expression of these transporters may contribute to the platinum drug-resistance phenotype observed in these cell lines. Indeed, other studies have shown that oxaliplatin is a substrate for the influx transporters OCT1-3, albeit, substrate affinity for these membrane carriers varied between the different reports [12,32,33,34,35] The relevance of OCT3 in oxaliplatin uptake in colorectal cancer was further demonstrated by higher expression levels in tumor specimens as compared with normal tissue [36]. Since a high concentration of oxaliplatin was used for the accumulation experiments, here, it is unlikely that the hCTR1 transporter contributed to the reduced platinum drug accumulation [11]. Although clinical studies have shown a correlation between ERCC1 expression in patients treated with the oxaliplatin-5-fluorouracil combination (see e.g., [21]), available databases such as TCGA do not provide data on gene expression after patients have become resistant to oxaliplatin treatment, which prevents “validation” of our finding with clinical samples.

The lack of correlation between sensitivity to oxaliplatin and the formation of platinum DNA adducts may possibly be the result of cellular sequestration of the drug away from its target. Previously, it has been shown that the ATPases ATP7A and ATP7B play a role in intracellular trafficking and efflux of the drug [10]. The efflux transporter ATP7A was significantly correlated to the formation of platinum DNA adducts and might, therefore, determine the formation of DNA adducts. Next to reduced accumulation and drug sequestration, platinum DNA adducts tolerance [7] might also play a role in drug resistance, since no reduction in DNA adduct formation was observed in oxaliplatin-resistant LoVo-Li cells or in the resistant ovarian cancer cell line, A2780/4OHP.

To gain insight into the alternative mechanisms of oxaliplatin resistance, we also performed gene expression arrays and subjected the data to ingenuity pathway analysis (IPA) to search for canonical pathways that were significantly affected. Several pathways were frequently and significantly affected and might add to the resistance mechanisms described above. The most frequently altered gene in the aryl hydrocarbon receptor (AHR) signaling pathway was ALDH1L2; this gene was decreased in all oxaliplatin resistant cell lines and is the mitochondrial homolog of ALDH1L1 which has been implicated in cell proliferation and apoptosis [37,38]. Another family member, ALDH1A1, was increased in five out of six drug-resistant cell lines and is a marker for cancer stem cells and chemoresistance [39].

In the p53 signaling pathway, the most frequently decreased genes were the proapoptotic genes, BAX and BBC3 (PUMA), which were reported previously to play a role in oxaliplatin resistance [22,23] and induction of apoptosis [40], respectively. Previously it was demonstrated that oxaliplatin decreased BIRC5 (survivin) levels [41,42]. Increased BIRC5 expression could, therefore, contribute to the observed oxaliplatin resistance. Similarly, increased expression of the inhibitor of apoptosis BIRC4 (XIAP) could also contribute to oxaliplatin resistance and counteract the increased expression of the cell death execution caspases 6 and 7 in the platinum-resistant ovarian cancer cell lines. These data are in line with the decrease in annexin A3 in l-OHP resistant colon cancer cells [43], as well as an activation of mutated p53 in l-OHP resistant ovarian cancer cells [44] and a change in multiple apoptosis regulating genes in resistant colon cancer cells [45].

Since the expression of ERCC1 is unaltered or even decreased in the drug-resistant cell lines, it is unlikely that nucleotide excision repair is involved in the resistance towards oxaliplatin in these cell lines. Decreased ERCC1 mRNA and protein expression in oxaliplatin resistant cells has been previously demonstrated [46] and was found to be correlated to cell cycle response rather than to DNA repair after platinum treatment. The role of BRCA1 in DNA damage response pathway was significantly changed, particularly in the LoVo-92/cOHP and LoVo-Li/4OHP cell lines. Increased expression of RAD51, Fanconi genes A, G, and M, and replication factor 2 and 5 suggest that homologous repair of double strand breaks could contribute to oxaliplatin resistance in these cell lines.

The xenobiotic metabolism pathways, highly significant in LoVo-Li/cOHP, suggested a possible contribution to oxaliplatin resistance of aldo-keto reductase family members, 1C1 and 1C3. Aldo-keto reductase family members have been implicated previously in poor prognosis and resistance towards platinum and other drugs [47,48,49]. Contrasting results were observed for the multidrug-resistant efflux transporter, ABCC2 (MRP2), which was decreased in LoVo-Li variants but increased in the A2780 variants, hence this ABC transporter does not appear to contribute consistently to oxaliplatin efflux. Previously, we compared 2008 ovarian cancer cells and an ABCC2 transfected variant for oxaliplatin sensitivity and observed a similar sensitivity of CDDP to that of 2008 cells with an IC50 of about 1.5 μM [28]. However, increased levels and defective glycosylation of MRP family members 1 and 4 in oxaliplatin resistant ovarian cancer cell lines were found to be associated with decreased platinum drug accumulation [50], while Myint et al. [51] observed that l-OHP was a substrate for MRP2 in isolated inverted MRP2 overexpressing vesicles.

Changes in gene expression observed in the drug-resistant cell lines as discussed above could be mediated by altered gene expression but might also be a result of gains and losses at the genomic level. Primarily, focal gains and losses were observed in the oxaliplatin resistant variants. The parental cell line LoVo-92 has been characterized by trisomy of chromosome (Chr) 5, 7 and 15 [52]. The major loss of Chr 15 in LoVo-92/4OHP suggests that the trisomy of Chr 15 was lost. In LoVo-92/cOHP only a partial loss of Chr 15 was observed. LoVo-Li which was derived from LoVo-92 also showed a gain of Chr 12p that was retained in the drug-resistant variants. A comparison of the aCGH profiles with mRNA expression data, using the program ACE-it, did not reveal common genes that were significantly altered both at the genomic and the mRNA level. Previously, it was shown that, in H69 small cell lung cancer cells, oxaliplatin induced three times more chromosomal alterations than that of cisplatin [53]. Since the resistant variants of the ovarian cancer A2780 cells harbor a much higher number of chromosomal aberrations, this suggests that these cells are more vulnerable to oxaliplatin than that of the colorectal cancer cell lines LoVo-92 and LoVo-Li. In the study with the H69 cells, it was shown that after removal of the drug and loss of the drug-resistant phenotype, many of the aberrations observed in the drug-resistant variants were lost but some were retained [53]. It was hence concluded that the similar changes found after treatment with cisplatin and oxaliplatin were independent of the drug-resistant phenotype.

One of the main findings of the present study is that sensitivity to oxaliplatin is highly correlated with total platinum accumulation which was associated with expression status of the organic cation transporters OCT1-3, hence highlighting the role of these influx transporters in acquisition of oxaliplatin resistance. Since formation of DNA adducts, which was highly correlated to ATP7A, was not related to oxaliplatin sensitivity, it can be concluded that mechanisms, other than DNA damage signaling, may contribute to the pharmacological activity of oxaliplatin. Decreased expression of proapoptotic genes such as Bax and BBC3(PUMA) and increased expression of inhibitors of apoptosis, as well as genes involved in homologous repair, warrants combination studies with drugs that target these proteins, to overcome resistance to oxaliplatin and other platinum drugs.

## 4. Materials and Methods

### 4.1. Chemicals

Oxaliplatin (l-OHP) was kindly provided by Sanofi-Synthelabo (Paris, France). Cisplatin (CDDP, Platinol^®^) was obtained from Bristol-Myers Squib B.V. (Woerden, The Netherlands) in a concentration of 0.5 mg/mL. The RPMI and DMEM culture medium were obtained from BioWhittaker (Cambrex BioSience, Verviers, Belgium) and fetal bovine serum (FBS) was obtained from GIBCO (Paisley, UK). All other chemicals were of analytical grade. Solutions were made in water purified by a Millipore Reagent Q system (Millipore, Bedford, OH, USA).

### 4.2. Cell Culture

The colon carcinoma cell lines, LoVo-92 and LoVo-Li, were kindly provided by Dr. Poupon [54]. LoVo-92 expresses wild type (wt) p53 and its derivative Lovo-Li has functionally inactive p53. The functional activity of LoVo-Li was determined by co-transformation of LoVo-Li cDNA with an ADE2 open reading frame under the control of a p53-responsive element into yeast. Yeast transformed with LoVo-Li cDNA resulted in 40% red colonies that did not express ADE2, while LoVo-92 resulted in 15% red colonies [54]. The p53 status was confirmed in our laboratory and was stable after trypsinization and further culturing [55]. The ovarian cancer cell line A2780 expresses wt-p53. For some comparative experiments we included the CDDP resistant variant of A2780, ADDP, which also has a p53 mutation [28]. This cell line is 41-fold resistant to CDDP and 7.5-fold cross-resistant to oxaliplatin as compared with the parent A2780 cells [28]. All cell lines were cultured in RPMI-1640 supplemented with 10% heat-inactivated FBS at 37 °C in a humidified atmosphere of 5% CO_2_ and tested by morphology check using microscope and growth curve analysis according to the Cell Line Verification Test Recommendations (ATCC-Technical Bulletin No. 8, 2008). Periodic assays were performed to check for mycoplasma contamination.

### 4.3. Establishment of Oxaliplatin Resistance

Resistance to oxaliplatin was induced by exposing LoVo-92, LoVo-Li, and A2780 cells to increasing concentrations of oxaliplatin using two different schedules for a period of 7 months. In the first schedule, cells were exposed to 4 h pulses every passage to mimic the clinical bolus treatment. In parallel, cells were exposed every passage for 72 h to mimic a continuous infusion. Platinum-resistant cell lines were termed after the parental cell line with 4OHP and cOHP extensions reflecting the resistance induction scheme. To maintain the resistance phenotype, cells were regularly exposed to oxaliplatin during routine culturing. For all mechanistic experiments as described below (growth inhibition, DNA adducts, drug uptake, gene expression, and microanalysis), cells were cultured without drug for at least one week.

### 4.4. Growth Inhibition Experiments

Growth inhibition experiments were performed at least three times using the sulforhodamine B (SRB) assay as previously described [56]. Briefly, cells were plated in triplicate in their specific culture medium (0.1 mL/well) in flat bottom 96 well plates (Costar, Cambridge, MA, USA) in densities related to their growth [57]. After 24 h, culture medium was added to control wells (0.1 mL/well), whereas, drug containing culture medium was added to the other wells and subsequently cultured for another 72 h. The drug concentration range used for CDDP was from 0.1 µM to 500 µM, whereas, that for oxaliplatin was 0.01 µM to 200 µM. To determine growth inhibition, cells were fixed with trichloroacetic acid (TCA) and stained with SRB protein dye. Optical density was measured at 540 nm and results were expressed relative to the control growth. A 50% growth inhibition concentration (IC_50_) was determined from the growth curves.

### 4.5. Oxaliplatin Accumulation and Formation of DNA Adducts

To determine cellular oxaliplatin accumulation and formation of DNA adducts, cells were exposed to either 20 μM l-OHP for 4 h or to 200 µM l-OHP for 24 h. To determine retention of the accumulated l-OHP and the formed DNA adducts, indicative for alterations in efflux or repair, cells were subsequently incubated for 3 h in a drug-free medium as described previously [28]. Since 200 μM OHP is a non-pharmacological concentration, which is not reached in patients, we also used 20 μM l-OHP at a shorter exposure for 4 h. The latter data were related to the accumulation at 200 μM. Cells were subsequently washed 3 times with ice-cold PBS and attached cells were harvested on ice by trypsinization, counted (for accumulation studies), and stored as cell pellets at −20 °C until analysis. For determination of accumulated l-OHP, cells were lysed in 0.5 mL 2 M NaOH and incubated overnight at 55 °C. Subsequently, 1 mL 1 M HCl was added and samples were measured using flameless atomic absorption spectrometry (FAAS, Varian SpectrAA-300 Zeeman Atomic Absorption Spectrometer) as described previously for CDDP [58] including a calibration curve ranging from 0.2 µM to 3.0 µM. To determine the formation of platinum-DNA adducts, DNA was isolated from the combined floating and attached cells using a QIAmp DNA Mini Kit (Westburg, Leusden, The Netherlands) and concentration was determined by measuring optical density at 260 nm and 280 nm (Nanodrop ND-1000, Isogen Life sciences BV, IJsselstein, The Netherlands). To DNA samples or Pt-standard solutions (ranging from 0.25 µM to 1.5 µM), 25 µL of 1.68 M NaCl was added to 250 µL, and samples were subsequently measured using FAAS, as described above.

### 4.6. Quantitative Gene Expression Measurement

To determine gene expression, RNA was isolated from cell pellets using Trizol (Invitrogen, Paisley, UK) according to the manufacturer’s protocol. After RNA quantification (nanodrop ND-1000), 500 ng to 1500 ng of RNA were used for cDNA synthesis, as described previously [59]. Subsequently, gene expression was quantified using TaqMan. To establish a calibration curve, human reference RNA (Stratagene, Amsterdam, The Netherlands) was used. The TaqMan gene expression assays were: Hs00427554_m1 (SLC22A1, OCT1), Hs00533907_m1 (SLC22A2, OCT2), Hs00222691_m1 (SLC22A3, OCT3), Hs00156229_m1 (hCTR1), Hs00163707_m1 (ATP7A), Hs00163739_m1 (ATP7B), Hs00157415_m1 (ERCC1), and Hs99999903_m1 the endogenous control human ACTB (ß-actin) [28].

### 4.7. Microarray Analysis of RNA and DNA

Parental cell lines and their oxaliplatin-resistant sublines were subjected to microarray analysis. Both array comparative genomic hybridization (aCGH) and mRNA expression arrays were performed. The isolation of genomic DNA (gDNA) for aCGH analysis was performed using the Wizard Genomic DNA Purification kit (Promega Benelux, Leiden, The Netherlands) according to the manufacturer’s protocol. Total RNA was isolated using Trizol extraction, as described above. The gDNA and RNA quality was checked (OD 260/280 > 1.8). Subsequently, aCGH was performed using the Agilent Human Genome CGH 44 K array platform (Agilent Technologies, Amstelveen, The Netherlands). Samples and human universal reference were hybridized using a cross array hybridization as described previously [60]. For mRNA expression, the Agilent Whole Genome Oligo Microarray 4 × 44K array platform was used according to the manufacturer’s protocol. Samples of drug-resistant cell lines were hybridized to their parental cell lines. The microanalyses were repeated twice.

### 4.8. Microarray Data Analysis

After hybridization and scanning of aCGH slides the raw data were extracted from the images using Agilent Feature Extraction (version 9.5.1.1). Subsequently, data were normalized and smoothened with the R-script “nowave” to increase accurate detection of breakpoints [61]. Genomic aberrations and breakpoints were determined using CGHcall which determines the probability of detected gains and losses [61]. For detection of copy number induced differential gene expression, CGHcall data and mRNA expression data were, subsequently, analyzed using ACE-it [62].

After hybridization and scanning of slides for genome wide gene expression, the raw data were extracted from the images using Agilent feature extraction and subsequently processed using the LIMMA package in Bioconductor to determine Lowess normalized Log2 ratios. For determination of enrichment of genes in canonical pathways, normalized Log2 ratios >1 or <−1 and *p* < 0.05 were analyzed using ingenuity pathway analysis (Ingenuity Systems, Redwood City, USA).

### 4.9. Statistical Analysis

Differences between cell lines regarding sensitivity, drug accumulation, adduct formation, and gene expression were evaluated using the Student *t*-test for paired and unpaired samples, provided by the statistical option of Excel. *p*-Values below 0.05 were considered as statistically significant. Correlation studies, both the parametric Pearson correlation coefficient (r) for linear correlation and the nonparametric Spearman’s rank correlation coefficient (rho), were performed using SPSS 16.0. Data analysis of the microarray data were performed with specific programs as described in the microarray sections.

## Figures and Tables

**Table 1:** Sensitivity to oxaliplatin and cisplatin in parental- and drug-resistant variants of colon and ovarian cancer cell lines.

Cell Line	l-OHP (µM)	CDDP (µM)	RF (l-OHP)	RF (CDDP)
LoVo-92	0.21 ± 0.04	1.50 ± 0.40		
LoVo-92/4OHP	1.69 ± 0.25	2.50 ± 1.31	7.9	1.7
LoVo-92/cOHP	2.51 ± 0.25	4.03 ± 1.64	11.8	2.7
LoVo-Li	0.75 ± 0.08	4.23 ± 1.65		
LoVo-Li/4OHP	3.03 ± 0.64	4.65 ± 0.35	4.0	1.1
LoVo-Li/cOHP	4.23 ± 0.92	7.57 ± 1.23	5.6	1.8
A2780	0.32 ± 0.03	0.50 ± 0.14		
A2780/4OHP	1.73 ± 0.40	3.50 ± 2.25	5.3	7.0
A2780/cOHP	3.61 ± 0.33	4.60 ± 0.70	11.1	9.3
ADDP	2.43 ± 0.35	20.3 ± 2.7	7.5	40.9

Colon (LoVo) and ovarian (A2780) cancer cell lines were made resistant by weekly 4 h (4OHP) and 72 h (cOHP) exposure to increasing concentrations of oxaliplatin. ADDP is an A2780 variant with acquired resistance to CDDP; data are from [28]. The IC_50_ values and resistance factors (RF) were determined after 72 h exposure to l-OHP and CDDP. Values are given in μM and are means ± SEM of at least three separate experiments.

**Table 2:** Correlation between quantitative gene expression data with total platinum accumulation, DNA adduct formation, and sensitivity to oxaliplatin.

		Pearson Correlation			Spearman’s Rho		
		Total Pt	Pt-DNA	IC_50_	Total Pt	Pt-DNA	IC_50_
Total Pt	Correlation		0.760 *	−0.667 *		0.533	−0.700 *
	*p*-value		0.018	0.050		0.139	0.036
Pt-DNA	Correlation	0.760 *		−0.398	0.533		−0.283
	*p*-value	0.018		0.289	0.139		0.460
OCT1	Correlation	0.882 *	0.841 *	−0.557	0.867 *	0.733 *	−0.467
	*p*-value	0.007	0.005	0.119	0.002	0.025	0.205
OCT2	Correlation	0.802 *	0.152	0.638	0.714	−0.214	−0.821 *
	*p*-value	0.030	0.745	0.123	0.071	0.645	0.023
OCT3	Correlation	0.949 *	0.688	−0.675	0.771	0.029	−0.829 *
	*p*-value	0.004	0.131	0.141	0.072	0.975	0.042
CTR1	Correlation	0.475	0.145	−0.413	0.762 *	−0.071	−0.619
	*p*-value	0.234	0.732	0.269	0.028	0.867	0.102
ATP7A	Correlation	−0.489	−0.494	−0.107	−0.644	−0.745 *	0.259
	*p*-value	0.182	0.177	0.784	0.061	0.021	0.500
ATP7B	Correlation	0.351	−0.054	−0.588	0.517	−0.217	−0.617
	*p*-value	0.354	0.890	0.096	0.154	0.576	0.077
ERCC1	Correlation	0.572	0.672 *	−0.457	0.500	0.650	−0.367
	*p*-value	0.108	0.048	0.216	0.170	0.058	0.332

Pearson and Spearman correlation analysis were performed between gene expression of transporters and DNA repair genes with total platinum accumulation, platinum DNA adducts formation, and sensitivity to oxaliplatin. The correlation coefficient and *p*-value are depicted. Significant correlations are marked with *.

**Table 3:** Significance of most frequently observed enriched canonical pathways.

	LoVo-92		LoVo-Li		A2780	
IPA Pathway	4OHP	cOHP	4OHP	cOHP	4OHP	cOHP
Axonal guidance signaling	3.16			2.35	3.56	1.92
Aryl hydrocarbon receptor signaling		2.81		3.95	2.32	3.06
p53 Signaling			1.71		2.90	3.84
Colorectal cancer metastasis signaling	2.72				4.10	2.97
ILK signaling	1.99				3.44	2.57
RAR activation	2.17	2.42		2.11		2.66
Role of macrophages, fibroblasts and endothelial cells in rheumatoid arthritis		2.00		2.98	3.93	2.04
Virus entry via endocytic pathways		1.78		3.08		3.28
Agrin interactions at neuromuscular junction		1.84				2.71
Cardiac hypertrophy signaling	2.00				2.02	
Caveolar-mediated endocytosis signaling		1.71				2.89
Cysteine metabolism			1.42		2.32	1.89
Glycine, serine and threonine metabolism			1.48		3.38	2.96
Hepatic fibrosis/hepatic stellate cell activation					2.94	2.32
Molecular mechanisms of cancer					3.51	2.43
p38 MAPK signaling		1.52			3.01	1.95
Semaphorin signaling in neurons	2.45				2.84	
Sphingosine-1-phosphate signaling					3.11	2.06
Starch and sucrose metabolism	1.77		1.40	4.36		
Tyrosine metabolism	1.83	1.75		2.37		
Role of BCRA1 in DNA damage response		2.30	5.76			
CXCR4 Signaling	2.42					
Xenobiotic metabolism by cytochrome P450				9.41		
Cell cycle: G1/S checkpoint regulation			4.63			
Xenobiotic metabolism signaling				5.57		

Global gene expression array data showing two-fold increased or decreased expression in the resistant cell lines relative to their parental cell lines were subjected to ingenuity pathway analysis (IPA). Most frequently observed enriched pathways are shown and depicted values are Log(*p*) determined with the Fisher exact test.

**Table 4:** Several common changes in the resistant cell lines in the expression of genes involved in induction and inhibition of apoptosis.

Decreased Proapoptotic Genes	Increased “Inhibition of Apoptosis” Genes
Bax	PCNA (replication)
PUMA	DNA damage response
Apaf1	IAP
Tumor suppressor serpin B5	Survivin
	BRCA1 in DNA damage

**Table 5:** Common or overlapping chromosomal aberrations.

Chr	Chr. Band	Probe Position	Aberration	Cell Lines
2	q37.1	232396451–233411853	Focal gain	LoVo-Li/4OHP, LoVo-Li/cOHP
4	p16.3–16.1	6503780–880103	Gain	A2780/4OHP, A2780/cOHP
7	q31.1	110361335–110795919	Focal loss	LoVo-Li/4OHP, LoVo-Li/cOHP
10	q21.3	69418458–69551488	Focal loss	A2780/4OHP, A2780/cOHP
12	q24.23–q24.31	120316650–120358330	Focal gain	LoVo-Li/4OHP, LoVo-Li/cOHP
15	q22–q26.2	66499644–98087372	Loss	LoVo-92/4OHP, LoVo-92/cOHP
16	p13.3	3427264–4180609	Focal gain	LoVo-Li/4OHP, LoVo-Li/cOHP, A2780/4OHP
17	q21.2	39782285–39993938	Focal gain	LoVo-Li/4OHP, LoVo-Li/cOHP, A2780/cOHP
19	p13.3	232080–637653	Focal gain	A2780/4OHP, A2780/cOHP
19	p13.11	17268193–17536526	Focal gain	LoVo-Li/4OHP, LoVo-Li/cOHP

Acquired chromosomal aberrations after induction of resistance were determined with aCGH analysis. Size and location of the aberrations were determined using CGHcall. Depicted are the common or overlapping gains and losses with the chromosome (Chr) number, chromosomal band (Chr band), and genomic position (probe position), and the cell lines in which the aberrations were observed. Focal gains and losses are defined as <3 Mb.

## Supplementary Materials

Supplementary materials can be found at https://www.mdpi.com/1422-0067/20/15/3619/s1.

## Abbreviations

aCGH	Array comparative genomic hybridization
CDDP	Cisplatin
hCTR	Human copper transporter
FBS	Fetal bovine serum
l-OHP	Oxaliplatin
MMR	Mismatch repair
NER	Nucleotide excision repair
OCT	Organic cation transporter
Pt	Platinum
SRB	Sulforhodamine B
