# Supplementary material for: Coexisting Molecular Determinants of Acquired Oxaliplatin Resistance in Human Colorectal and Ovarian Cancer Cell Lines
Source: Int J Mol Sci. 2019 Jul 24;20(15):3619. doi: 10.3390/ijms20153619 (PMC6696456; doi:10.3390/ijms20153619)
Supplement: Supplementary file 1 [file ijms-20-03619-s001.pdf]

Supplementary Materials

Table S1. Gene expression of altered genes in the most relevant pathways as determined with Ingenuity Pathway Analysis.

Genes in Pathway		LoVo-92		LoVo-Li		A2780		ADDP
		4OHP	cOHP	4OHP	cOHP	4OHP	cOHP	
AHR Signaling								
AHR	aryl hydrocarbon receptor	0.61	0.33	-0.57	0.19	-0.14	0.33	4.08
ALDH1A1	aldehyde dehydrogenase 1 ,A1	1.51	1.53	0.82	-0.99	2.37	4.64	6.19
ALDH1A3	aldehyde dehydrogenase 1, A3	-0.02	-0.33	-0.66	-0.03	0.03	-0.26	1.54
ALDH1L2	aldehyde dehydrogenase 1, L2	-2.54	-3.33	-3.45	-2.83	-2.24	-3.67	-1.26
ALDH3A2	aldehyde dehydrogenase 3, A2	-0.40	-0.30	0.31	1.20	0.14	0.24	1.08
ALDH6A1	aldehyde dehydrogenase 6, A1	0.15	0.52	-1.89	-1.25	-1.36	-1.90	-0.36
Apaf1	apoptotic peptidase activating factor 1	-0.16	0.01	0.54	1.27	-1.09	-1.16	-0.60
ATR	ataxia telangiectasia and Rad3 related	0.49	0.34	0.40	-0.16	1.20	0.79	0.73
BAX	BCL2-associated X protein	-0.91	-1.20	0.84	-0.38	-1.60	-1.12	-2.07
CCNA2	Cyclin A2	0.46	0.88	1.70	0.09	0.87	1.07	0.58
CCND1	Cyclin D1	-1.06	-0.49	1.24	1.10	-0.17	0.00	0.04
CCND2	Cyclin D2	-0.08	-0.15	-0.20	-0.29	-0.18	-0.10	0.99
CCND3	Cyclin D3	-0.14	0.09	1.50	0.42	1.14	0.76	-0.43
CCNE2	Cyclin E2	0.72	0.63	0.84	-0.20	0.52	0.06	-0.26
CDK6	Cyclin-dependent kinase 6	0.57	0.33	0.52	0.61	2.02	3.75	0.39
CDKN1A/p21	cyclin dependent kinase inhibitor 1A	-0.31	-0.77	-0.88	-0.45	-0.43	-0.78	-2.73
CDKN2A/p16	cyclin dependent kinase inhibitor 2A	-0.59	-1.19	0.34	0.59	0.78	0.67	1.87
CHEK1	CHK1 checkpoint homolog	0.68	1.05	0.59	-0.41	0.59	0.66	-0.20
CHEK2	CHK2 checkpoint homolog	0.04	0.32	0.52	0.02	0.46	0.26	-1.28
CYP1B1	cytochrome P450, family 1, B1	0.60	1.26	0.29	0.26	1.73	1.03	0.29
E2F1	E2F transcription factor 1	0.99	1.12	2.40	0.49	0.48	0.71	-1.15
FAS	**Fas,** TNF receptor superfamily, member 6	0.78	0.25	0.11	-0.87	-0.24	-2.71	-1.50
FOS	v-fos FBJ murine osteosarcoma viral oncogene homolog	0.08	-0.60	0.54	0.50	0.42	-1.24	-1.15
GSTA2	gluthation-s-tranferase A2	-0.09	0.17	0.24	0.26	-1.54	-0.60	-1.75
GSTA4	gluthation-s-tranferase A4	0.59	0.08	-0.85	-1.63	-0.12	-1.13	0.29
GSTA5	gluthation-s-tranferase A5	0.13	0.10	0.23	0.18	-1.98	-0.82	-1.90
GSTM1	gluthation-s-tranferase M1	-0.34	-0.23	0.36	0.38	0.11	-0.97	-1.78
GSTM2	gluthation-s-tranferase M2	-0.13	-0.24	-0.09	0.24	0.62	0.41	-2.12
GSTM3	gluthation-s-tranferase M3	0.14	0.21	0.54	-0.10	-0.18	-3.32	-3.72
GSTT2	gluthation-s-tranferase T2	-0.30	-0.03	0.34	-0.04	0.15	0.71	-2.85
HSP90AA1	heat shock protein 90kDa alpha, A1	0.48	-0.79	1.96	-0.08	0.36	0.03	-1.18
HSPB1	heat shock 27kDa protein 1	-0.13	-0.21	0.91	0.69	2.17	-2.64	-2.71
HSPB3	heat shock 27kDa protein 3	-0.24	-1.25	-0.18	-0.40	0.00	2.09	0.23
IL1A	interleukin 1, alpha	0.02	0.11	0.15	1.28	0.06	-0.05	-0.04
IL1B	interleukin 1, beta	0.79	2.76	0.20	3.04	-0.07	0.01	-0.04
IL6	interleukin 6	0.09	-0.08	0.11	0.04	0.40	-2.49	-2.95
JUN	jun oncogene	0.09	1.09	-1.00	0.88	0.64	3.19	3.99
MAPK3	mitogen-activated protein kinase 3	-1.03	-0.84	0.90	1.19	-1.33	-0.87	-0.26
MGST1	microsomal glutathione S-transferase 1	0.67	0.75	0.70	0.35	1.13	1.77	1.39
MGST2	microsomal glutathione S-transferase 2	0.38	0.11	-0.14	-0.47	0.03	1.63	-0.04
NCOA7	nuclear receptor coactivator 7	0.53	0.47	-0.60	0.11	-0.51	0.18	1.56
NCOR2	nuclear receptor co-repressor 2	-0.60	-0.75	0.35	1.41	-0.44	0.29	-0.01
NFIA	nuclear factor I A	0.40	0.55	-0.09	-0.40	4.23	0.50	1.86
NFIB	nuclear facor I B	0.15	0.05	0.20	0.37	3.33	3.74	4.17
NFKB2	nuclear factor kappa light polypeptide gene enhancer in B-cells 2	-0.48	-0.30	-0.27	0.25	-1.42	-0.97	-0.43

NR2F1	nuclear receptor sub family 2, F 1	0.32	0.22	1.16	1.03	0.78	-1.63	-1.09
NRIP1	nuclear receptor Interacting protein 1	0.44	0.19	0.10	-0.16	-0.60	-1.90	-2.89
NQO1	NAD(P)H dehydrogenase, quinone 1	0.51	1.63	-0.20	0.09	0.91	0.83	0.09
RARB	retinoic acid receptor, beta	0.05	-0.23	-0.29	-0.21	1.46	2.29	3.45
RXRB	retinoid X receptor, beta	-1.91	-1.75	0.34	0.77	-0.75	-0.80	0.03
SMARCA4	SWI/SNF related matrix associated, actin dependent regulator of chromatin, a 4	-0.57	-0.49	1.18	1.11	0.30	0.56	0.68
SRC	v-src sarcoma viral oncogene homolog	0.31	0.35	-1.59	-0.65	-0.19	-1.01	-0.10
TGFB1	transforming growth factor, beta 1	-0.69	-0.75	0.51	0.82	-1.24	-0.25	-1.83
TGFB2	transforming growth factor, beta 2	0.23	0.62	0.41	0.43	-0.78	0.47	-0.37
TGFB3	transforming growth factor, beta 3	-0.21	-0.01	0.17	0.05	1.12	1.34	0.72
TP53	tumor protein p53	-0.45	-0.37	0.05	0.63	-1.29	-1.66	-0.10
p53 Signaling								
AKT1	v-Akt murine thymoma viral oncogene homolog 1	-0.64	-0.70	0.27	-0.25	-0.15	-0.16	-1.57
AKT3	v-Akt murine thymoma viral oncogene homolog 3	-0.09	-0.20	0.08	0.21	1.79	0.16	-0.31
Apaf1	apoptotic peptidase activating factor 1	-0.16	0.01	0.54	1.27	-1.09	-1.16	-0.60
ATM	ataxia telangiectasia mutated	-0.04	-0.08	-0.04	0.66	-0.04	-0.27	1.26
ATR	ataxia telangiectasia and Rad3 related	0.49	0.34	0.40	-0.16	1.20	0.79	0.73
BAI1	brain-specific angiogenesis inhibitor 1	-0.45	-0.36	-0.63	-0.51	2.55	-1.06	-1.31
BAX	BCL2-associated X protein	-0.91	-1.20	0.84	-0.38	-1.60	-1.12	-2.07
BBC3/PUMA	BCL2 binding component 3	-0.79	-0.70	-1.07	-0.14	-1.11	-1.41	-0.79
Bcl2	B-cell CLL/Lymphoma 2	0.02	-0.34	0.78	0.68	-1.71	-1.13	0.78
BCL2L1	BCL2-like 1	-0.06	-0.14	-1.17	-0.92	-0.28	-0.19	0.22
BIRC1/NIAP	baculoviral IAP repeat-containing 1	-0.08	-0.18	-0.08	0.06	-0.27	-0.13	0.44
BIRC4/XIAP	baculoviral IAP repeat-containing 4	0.51	0.54	-1.13	-0.39	0.58	0.00	-0.20
BIRC5/Survivin	baculoviral IAP repeat-containing 5	0.02	0.07	1.36	0.29	0.19	0.93	-3.47
C12ORF5	chromosome 12 open reading frame 5	0.44	0.15	1.03	-0.24	-0.18	-0.014	-1.22
CASP6	caspase 6	0.00	0.52	0.83	0.11	0.19	1.06	1.18
CASP7	caspase 7	0.11	0.29	0.54	0.42	1.16	1.84	1.40
CASP10	caspase 10	-0.06	-0.26	0.53	0.15	-2.80	-1.72	1.53
CCND1	Cyclin D1	-1.06	-0.49	1.24	1.10	-0.17	0.00	0.04
CCND2	Cyclin D2	0.01	0.00	0.16	0.00	-0.42	-0.08	-0.02
CCNG1	Cyclin G1	0.12	-0.42	-1.00	-0.96	-0.40	-0.38	-0.01
CDK2	Cyclin-dependent kinase 2	-0.02	0.05	0.33	0.06	-0.20	0.17	-0.01
CDK4	Cyclin-dependent kinase 4	-0.48	-0.18	1.02	-0.07	0.13	0.15	-0.31
CDKN1A/p21	cyclin dependent kinase inhibitor 1A	-0.31	-0.77	-0.88	-0.45	-0.43	-0.78	-2.73
CDKN2A/p16	cyclin dependent kinase inhibitor 2A	-0.59	-1.19	0.34	0.59	0.78	0.67	1.87
CHEK1	CHK1 checkpoint homolog	0.68	1.05	0.59	-0.41	0.59	0.66	-0.20
CHEK2	CHK2 checkpoint homolog	0.04	0.32	0.52	0.02	0.46	0.26	-1.28
E2F1	E2F transcription factor 1	0.99	1.12	2.40	0.49	0.48	0.71	-1.15
FAS	**Fas,** TNF receptor superfamily, member 6	0.78	0.25	0.11	-0.87	-0.24	-2.71	-1.50
GADD45A	growth arrest and DNA damage inducible alpha	-0.28	0.55	-0.72	0.74	-1.91	-1.78	0.52
GADD45B	growth arrest and DNA damage inducible beta	-0.53	-0.58	-0.66	1.08	-1.10	-0.52	0.85
HDAC1								
HIPK2	homeodomain interacting protein kinase 2	0.30	-0.16	-0.36	-0.11	2.60	2.14	1.87
JMY	junction mediating and regulatory protein, p53 cofactor							
JUN	JUN oncogene	0.09	1.09	-1.00	0.88	0.64	3.19	3.99
LRDD	leucine-rich repeats and death domain containing	-0.70	-0.66	0.33	0.22	0.08	-0.34	-1.89
PCNA	proliferating cell nuclear antigen	0.72	0.90	2.20	-0.37	1.34	1.23	-0.65
PERP	TP53 apoptosis effector	-0.35	-0.54	-0.30	-0.73	-1.65	-1.21	-0.40
PIK3C2G	phosphoinositide-3-kinase, class 2, gamma polypeptide	0.03	0.09	0.03	0.10	0.19	0.36	3.25

PIK3CA	phosphoinositide-3-kinase, catalytic, alpha polypeptide	0.14	0.14	-0.60	-0.45	-1.35	-1.67	-1.67
PIK3R1	phosphoinositide-3-kinase, catalytic, beta polypeptide	0.24	0.31	0.20	-0.36	0.53	1.49	0.32
PLAGL1	pleiomorphic adenoma gene-like 1	-0.76	-0.85	-0.10	0.14	1.94	3.38	3.08
PMAIP1/NOXA	phorbol-12-myristate-13-acetate-induced protein 1	0.14	-0.10	-0.89	-0.20	2.14	0.92	3.08
SERPINE2	serpin peptidase inhibitor, clade2, 1	0.28	0.99	-0.35	0.48	-0.73	-2.08	-2.42
SERPINB5	serpin peptidase inhibitor, clade B (ovalbumin), member 5	-1.01	-1.89	-1.20	-0.81	0.09	0.10	-0.09
SFN	stratifin	-0.87	-0.46	0.64	0.15	-1.34	-1.29	-1.06
SNAI2	snail homolog 2	0.06	0.17	0.14	0.78	1.13	-1.92	-3.92
TNFRSF10A	tumor necrosis factor receptor superfamily, member 10a	0.21	-0.03	-0.18	-0.25	2.35	0.17	0.10
TNFRSF10B	tumor necrosis factor receptor superfamily, member 10b	-0.03	0.22	-1.12	-0.01	-0.70	-0.64	-0.69
TP53	tumor protein p53	-0.45	-0.37	0.05	0.63	-1.29	-1.66	-0.10
TP53I3	tumor protein p53 inducible protein 3	-0.22	0.08	1.31	-0.33	-0.10	-0.36	0.13
TP53INP1	tumor protein p53 inducible nuclear protein 1	0.20	-0.97	-1.13	-1.62	-0.47	-2.93	-1.41
BRCA1 in DNA damage response								
BARD1	BRCA1 associated RING domain 1	0.13	0.89	1.18	0.74	0.28	0.38	0.69
BLM	Bloom syndrome	0.58	1.15	1.06	0.19	0.41	0.61	-1.01
BRCA1	breast cancer 1	0.30	0.90	0.75	-0.06	-0.17	-0.21	-0.40
BRCA2	breast cancer 2	0.34	0.77	0.64	-0.09	0.29	0.41	0.32
CHEK1	CHK1 checkpoint homolog	0.68	1.05	0.59	-0.41	0.59	0.66	-0.20
E2F1	E2F transcription factor 1	0.99	1.12	2.40	0.49	0.48	0.71	-1.15
E2F3	E2F transcription factor 3	0.15	0.16	1.04	0.23	1.03	0.96	0.03
E2F4	E2F transcription factor 4	-0.75	-0.39	1.29	0.96	0.00	0.12	-0.32
E2F5	E2F transcription factor 5	-0.06	-0.85	-1.37	0.04	-0.43	-0.39	-0.34
FANCA	Fanconi anemia complementation group A	0.26	0.71	1.80	0.35	1.50	1.78	-0.22
FANCG	Fanconi anemia complementation group G	-0.37	0.34	1.43	0.74	-0.06	-0.05	-0.68
FANCM	Fanconi anemia complementation group M	0.60	0.51	1.01	0.10	0.07	0.24	-0.03
PLK1	polo-like kinase 1 (Drosophila)	-0.24	-0.04	1.81	0.78	-0.44	-0.24	-0.14
RAD18	RAD18 homolog	0.82	1.02	1.15	0.29	0.54	0.24	0.44
RAD51	RAD51 homolog	0.39	1.12	1.58	0.32	0.42	0.50	-0.56
RFC2	replication factor C 2	0.23	1.26	1.44	0.25	0.77	0.71	0.96
RBL1	retinoblastoma-like 1 (p107)	0.32	0.62	1.39	0.12	0.63	0.55	0.34
RFC5	replication factor C 5	0.08	0.62	1.53	0.14	0.62	0.93	-0.17
SLC19A1	solute carrier family 19 (folate transporter), member 1	0.32	0.45	1.38	0.66	0.37	0.79	0.48
SMARCA4	SWI/SNF related matrix associated, actin dependent regulator of chromatin, a 4	-0.57	-0.49	1.18	1.11	0.30	0.56	0.68
SMARCD2	SWI/SNF related matrix associated, actin dependent regulator of chromatin, a 4	-1.90	-1.53	1.62	1.47	-0.61	0.44	0.00
Xenobiotic Metabolism Signaling								
ABCB1	ATP-binding cassette, sub-family B (MDR/TAP), member 1	0.47	0.95	1.68	2.12	0.02	0.08	0.16
ABCC2	ATP-binding cassette, sub-family C (MDR/TAP), member 2	0.13	0.40	-1.73	-2.92	1.94	1.76	5.15
ALDH1A1	aldehyde dehydrogenase 1 ,A1	1.51	1.53	0.82	-0.99	2.37	4.64	6.19
ALDH3A2	aldehyde dehydrogenase 3, A2	-0.40	-0.30	0.31	1.20	0.14	0.24	1.08
ALDH6A1	aldehyde dehydrogenase 6, A1	0.15	0.52	-1.89	-1.25	-1.36	-1.90	-0.36
CYP3A7	cytochrome P450, family 3, subfamily A, polypeptide 7	0.67	0.41	-0.56	-1.59	0.07	0.41	1.04
GSTA4	gluthation-s-tranferase A4	0.59	0.08	-0.85	-1.63	-0.12	-1.13	0.29

HMOX1	heme oxygenase (decycling) 1	-0.93	-0.14	0.93	1.20	1.72	0.96	-0.80
HRAS	v-Ha-ras Harvey rat sarcoma viral oncogene homolog	-0.59	-0.31	1.88	1.07	0.11	0.47	-0.32
IL1A	interleukin 1, alpha	0.02	0.11	0.15	1.28	0.06	-0.05	-0.04
IL1B	interleukin 1, beta	0.79	2.76	0.20	3.04	-0.07	0.01	-0.04
MAOB	monoamine oxidase B	-1.32	-2.09	-0.23	-1.55	0.13	0.32	0.01
MAPK3	mitogen-activated protein kinase 3	-1.03	-0.84	0.90	1.19	-1.33	-0.87	-0.26
NCOR2	nuclear receptor co-repressor 2	-0.60	-0.75	0.35	1.41	-0.44	0.29	-0.01
PRKCD	protein kinase C, delta	-0.70	-0.35	1.00	1.18	-0.09	-0.11	-0.17
SLC01B1/OATP2	solute carrier organic anion transporter family, member 1B1	0.52	-0.33	-1.09	-1.11	0.07	-0.28	-0.07
UGT2B7	UDP glucuronosyltransferase 2 family, polypeptide B7	0.35	0.35	-1.81	-1.42	0.05	-0.10	-0.17
UGT2B10	UDP glucuronosyltransferase 2 family, polypeptide B10	0.08	0.19	-1.52	-1.17	-0.27	-0.02	0.10
UGT2B11	UDP glucuronosyltransferase 2 family, polypeptide B11	0.32	0.10	-1.83	-1.65	0.10	-0.02	-0.04
UGT2B15	UDP glucuronosyltransferase 2 family, polypeptide B15	0.39	0.04	-1.80	-1.75	0.09	0.12	0.30
UGT2B28	UDP glucuronosyltransferase 2 family, polypeptide B28	0.06	-0.20	-1.48	-1.24	0.01	-0.34	0.00
UST	uronyl-2-sulfotransferase	0.50	-0.28	-1.62	-1.38	1.63	1.50	2.08
Metabolism of Xenobiotics by p450								
AKR1C1	aldo-keto reductase family 1, member A1 (aldehyde reductase)	2.59	2.72	0.37	1.48	-1.72	-0.19	0.60
AKR1C3	aldo-keto reductase family 1, member A3 (aldehyde reductase)	2.11	1.91	1.38	2.29	0.87	0.80	-0.75
CSGALNACT1	chondroitin sulfate N-acetylgalactosaminyltransferase 1	-0.84	-1.26	0.53	0.58	0.64	0.41	-1.09
CYP2J2	cytochrome P450, family 2, subfamily J, polypeptide 2	-0.03	0.37	0.39	1.05	0.53	0.17	4.29
CYP2S1	cytochrome P450, family 2, subfamily S, polypeptide 1	-0.03	0.37	-0.30	1.16	-0.16	0.06	-0.12
CYP3A7	cytochrome P450, family 3, subfamily A, polypeptide 7	0.67	0.41	-0.56	-1.59	0.07	0.41	1.04
CYP51A1	cytochrome P450, family 51, subfamily A, polypeptide 1	1.00	-0.44	-0.56	1.24	-0.66	-1.22	0.24
DHDH	dihydrodiol dehydrogenase (dimeric)	-0.21	0.29	-0.84	-1.08	1.67	-0.19	3.74
DHRS9	dehydrogenase/reductase (SDR family) member 9	0.30	0.40	0.64	1.27	0.15	0.29	-0.25
DHRS2	dehydrogenase/reductase (SDR family) member 2	-0.58	-0.48	0.90	1.04	-2.61	1.17	0.41
GSTA4	gluthation-s-tranferase A4	0.59	0.08	-0.85	-1.63	-0.12	-1.13	0.29
LTC4S	leukotriene C4 synthase	-1.46	-1.86	-2.33	-1.19	0.25	-0.37	1.74
UGT2B7	UDP glucuronosyltransferase 2 family, polypeptide B7	0.35	0.35	-1.81	-1.42	0.05	-0.10	-0.17
UGT2B10	UDP glucuronosyltransferase 2 family, polypeptide B10	0.08	0.19	-1.52	-1.17	-0.27	-0.02	0.10
UGT2B11	UDP glucuronosyltransferase 2 family, polypeptide B11	0.32	0.10	-1.83	-1.65	0.10	-0.02	-0.04
UGT2B15	UDP glucuronosyltransferase 2 family, polypeptide B15	0.39	0.04	-1.80	-1.75	0.09	0.12	0.30
UGT2B28	UDP glucuronosyltransferase 2 family, polypeptide B28	0.06	-0.20	-1.48	-1.24	0.01	-0.34	0.00
Cell cycle G1/S Checkpoint Regulation								
CCND1	Cyclin D1	-1.06	-0.49	1.24	1.10	-0.17	0.00	0.04
CCND3	Cyclin D3	-0.14	0.09	1.50	0.42	1.14	0.76	-0.43
CDC25A	cell division cycle 25 homolog A (S. pombe)	0.24	0.49	1.85	0.79	0.65	0.57	-0.78
CDK2	Cyclin-dependent kinase 2	0.20	0.40	1.47	0.32	-0.19	0.72	-0.98
CDK4	Cyclin-dependent kinase 4	-0.48	-0.18	1.02	-0.07	0.13	0.15	-0.31
CDKN1B	cyclin-dependent kinase inhibitor 1B	0.10	0.26	-1.06	0.27	0.76	0.00	0.58

	(p27, Kip1)							
E2F1	E2F transcription factor 1	0.99	1.12	2.40	0.49	0.48	0.71	-1.15
E2F3	E2F transcription factor 3	0.15	0.16	1.04	0.23	1.03	0.96	0.03
E2F4	E2F transcription factor 4	-0.75	-0.39	1.29	0.96	0.00	0.12	-0.32
E2F5	E2F transcription factor 5	-0.06	-0.85	-1.37	0.04	-0.43	-0.39	-0.34
HDAC5	histone deacetylase 5	-0.63	-0.86	-1.10	-0.23	-1.02	-0.06	0.14
RBL1	retinoblastoma-like 1 (p107)	0.32	0.62	1.39	0.12	0.63	0.55	0.34
SUV39H1	suppressor of variegation 3-9 homolog 1 (Drosophila)	-0.08	0.18	1.19	0.19	0.27	0.36	-0.53
TFDP1	transcription factor Dp-1	-0.58	-0.41	1.45	0.94	0.26	0.33	-0.65
TGFB2	transforming growth factor, beta 2	0.23	0.62	0.41	0.43	-0.78	0.47	-0.37

Values are depicted as log2 ratios of the resistant cell line relative to the parental cell line.

Figure S1. Chromosomal profiles (normailized log2 ratio) of oxaliplatin resistant and parental cell lines. Profiles were established by CGHcall and depict log2 ratios and aberrant calls of resistant versus parental cell lines and parental cell lines versus ref DNA (ref xx and ref XY)

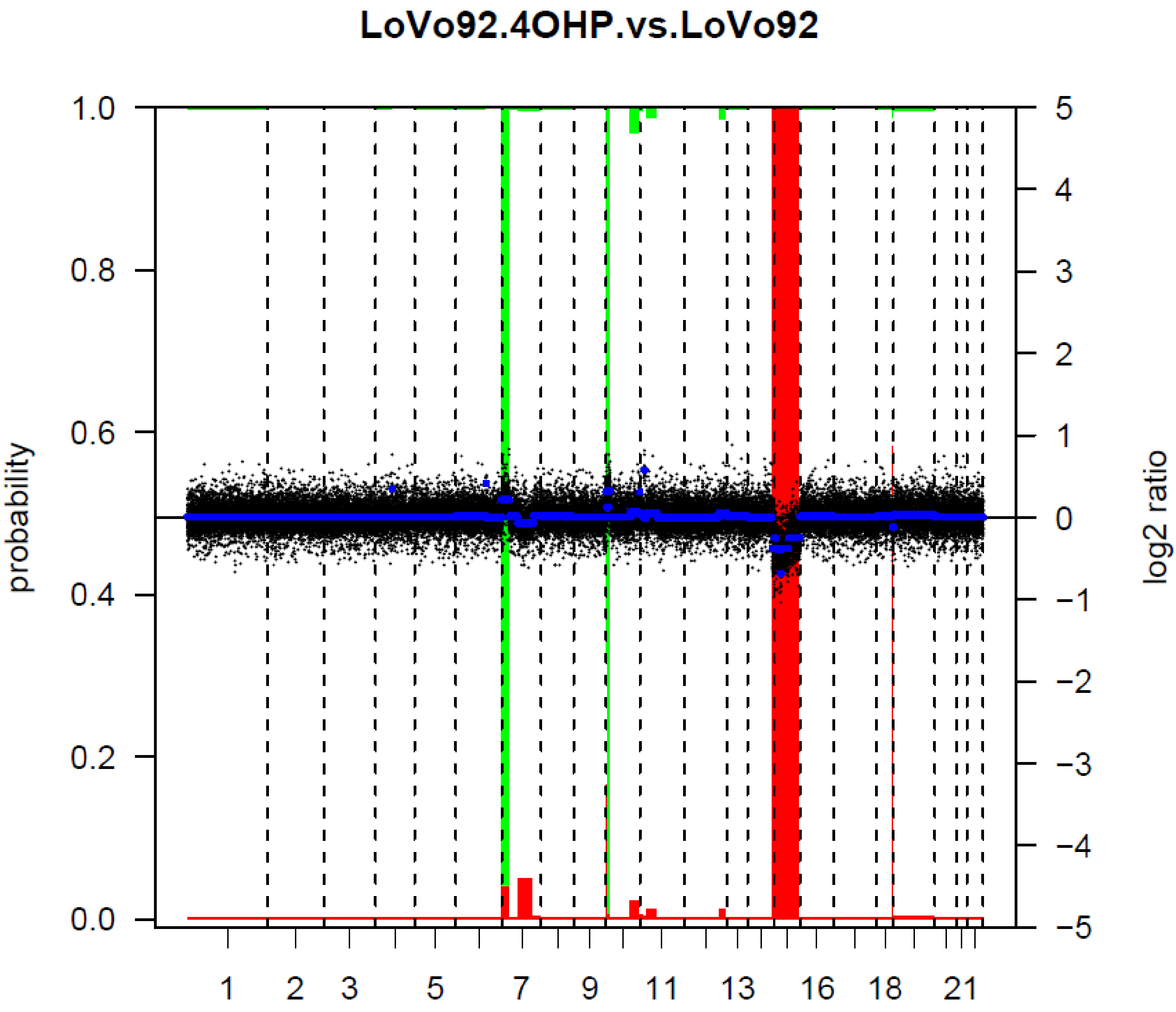

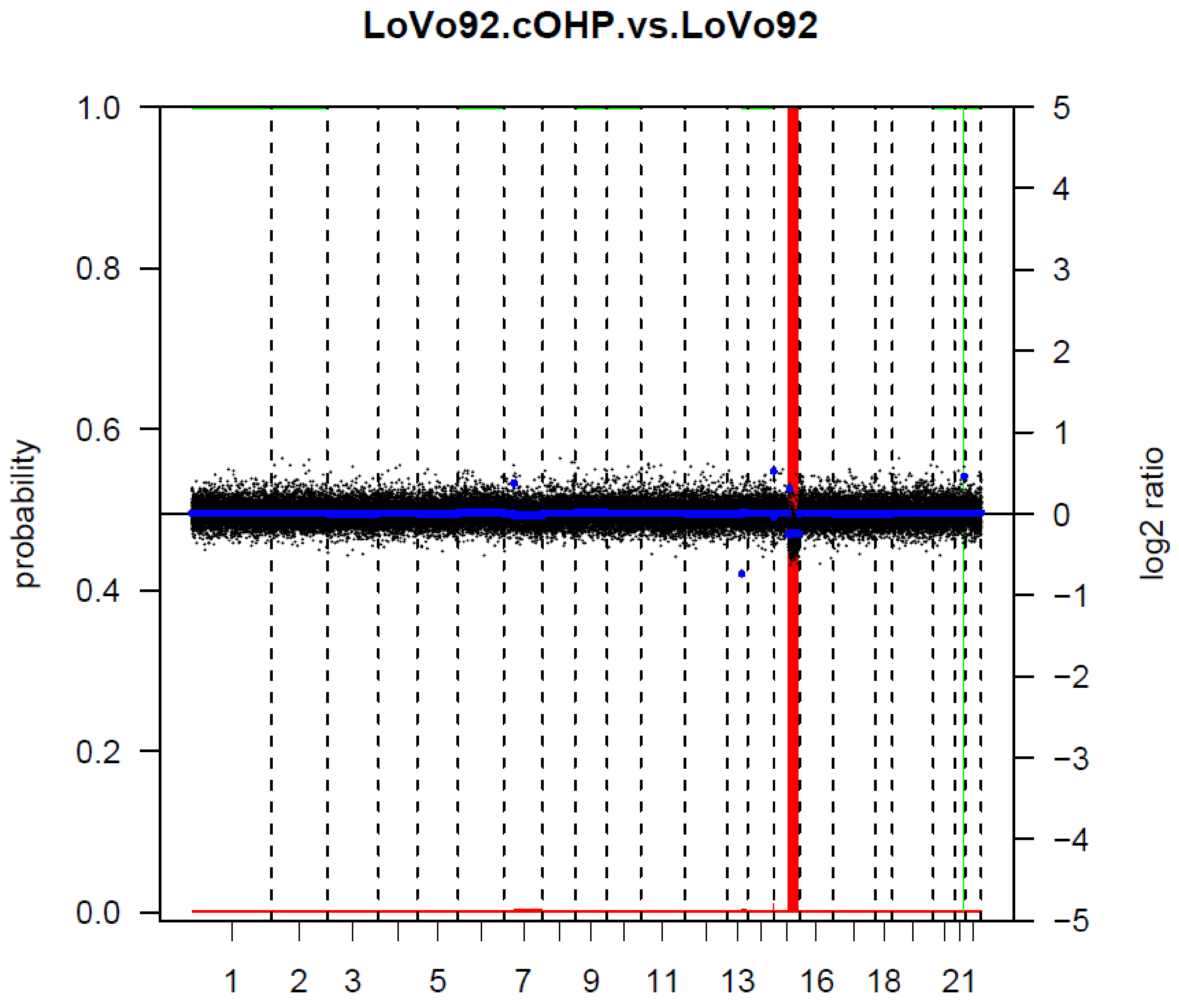
LoVo92.cOHP.vs.LoVo92
probability
log2 ratio
1.0
0.8
0.6
0.4
0.2
0.0
5
4
3
2
1
0
−1
−2
−3
−4
−5
1
2
3
5
7
9
11
13
16
18
21

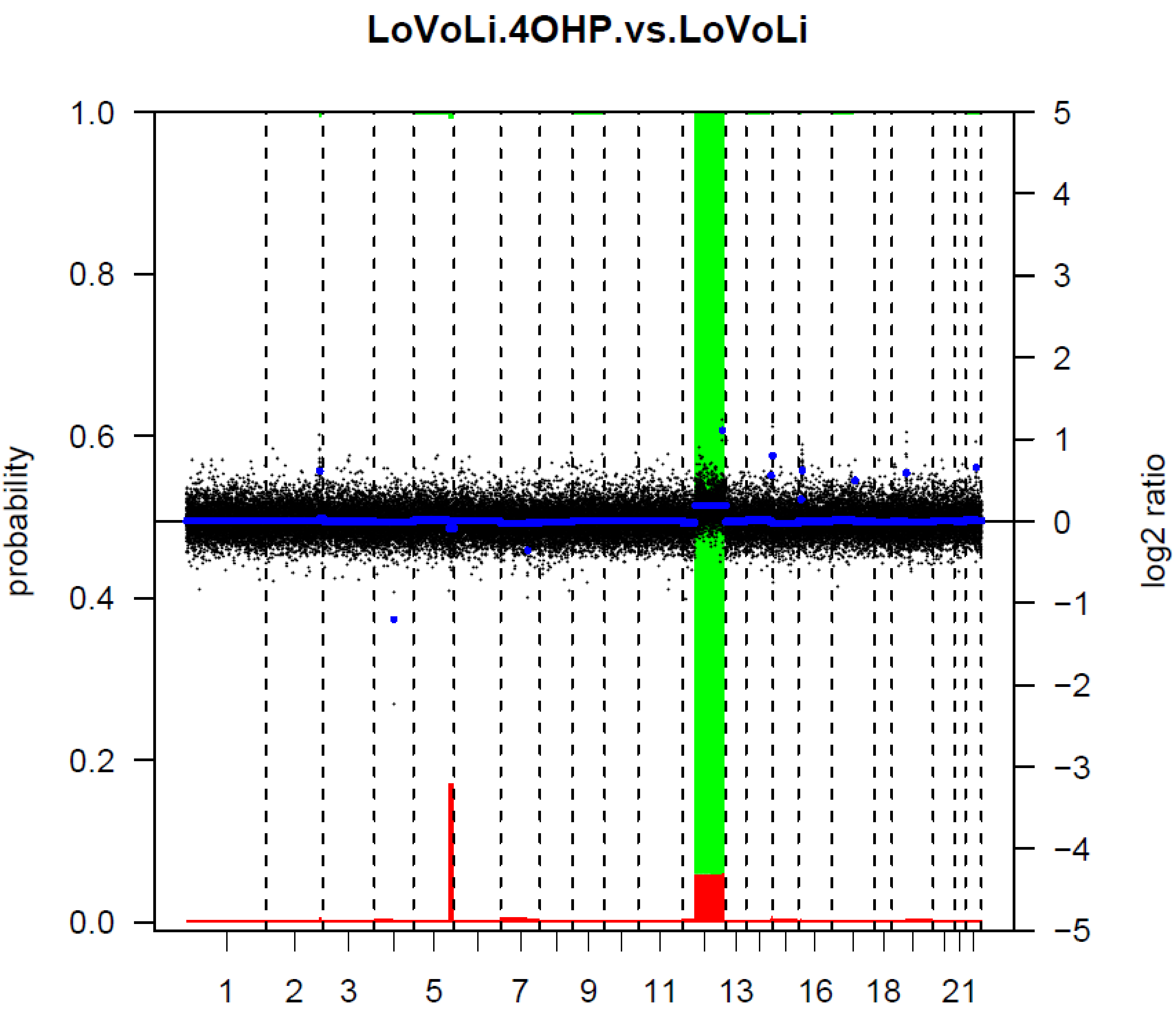

LoVoLi.4OHP.vs.LoVoLi
probability
log2 ratio
1.0
0.8
0.6
0.4
0.2
0.0
5
4
3
2
1
0
−1
−2
−3
−4
−5
1
2
3
5
7
9
11
13
16
18
21

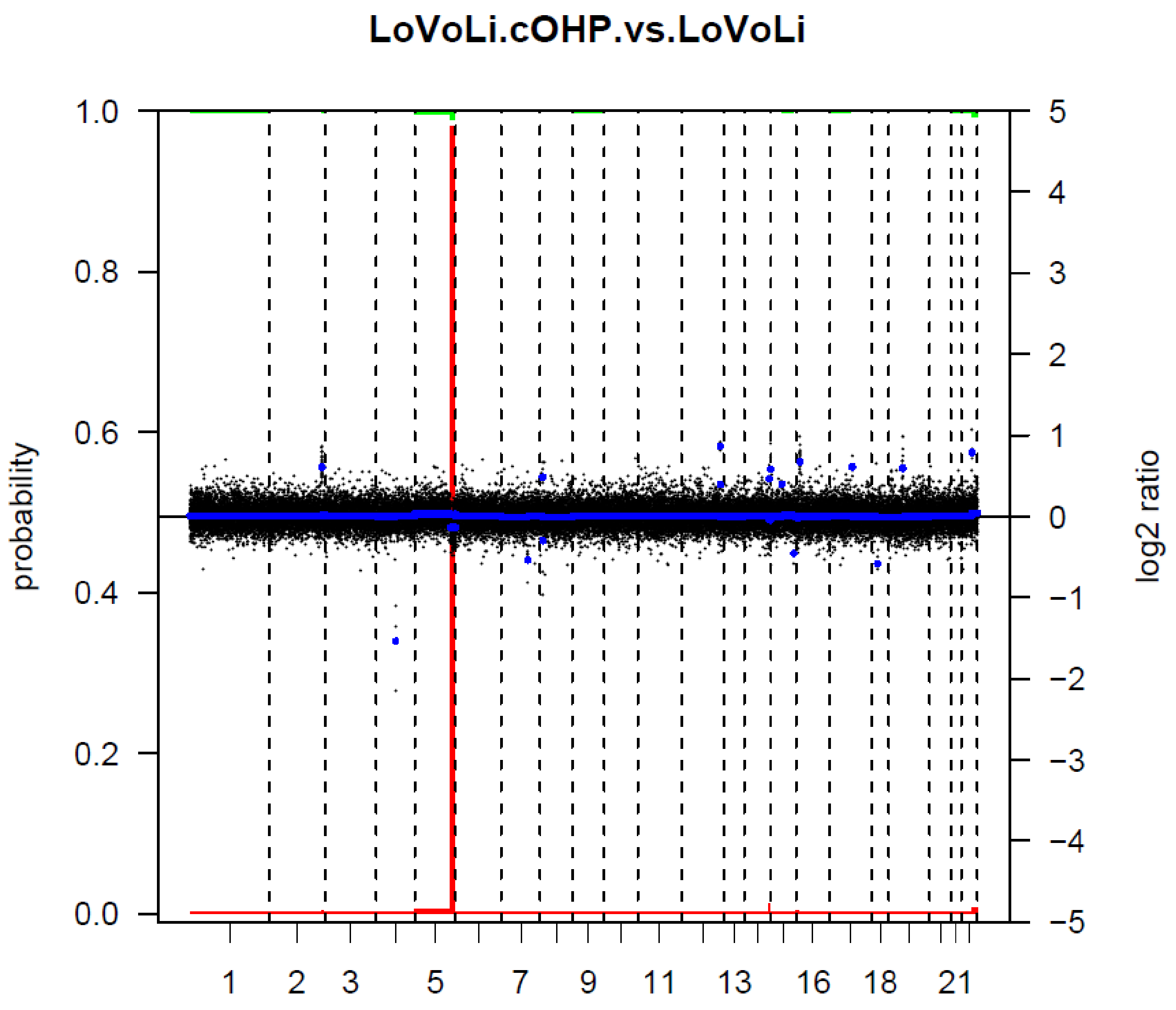
LoVoLi.cOHP.vs.LoVoLi
probability
log2 ratio
1.0
0.8
0.6
0.4
0.2
0.0
5
4
3
2
1
0
−1
−2
−3
−4
−5
1
2
3
5
7
9
11
13
16
18
21

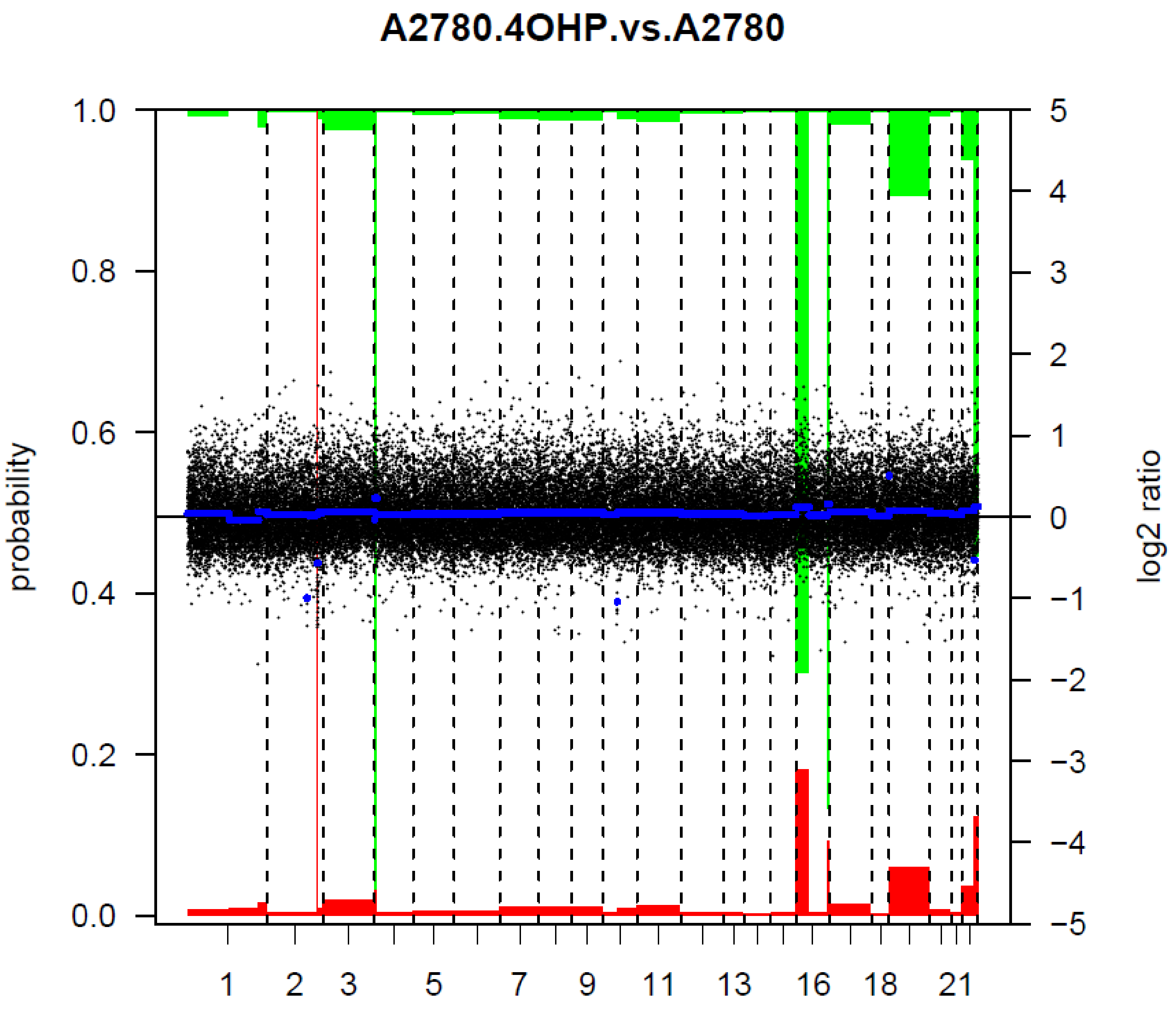
A2780.4OHP.vs.A2780
probability
log2 ratio
1.0
0.8
0.6
0.4
0.2
0.0
5
4
3
2
1
0
−1
−2
−3
−4
−5
1
2
3
5
7
9
11
13
16
18
21

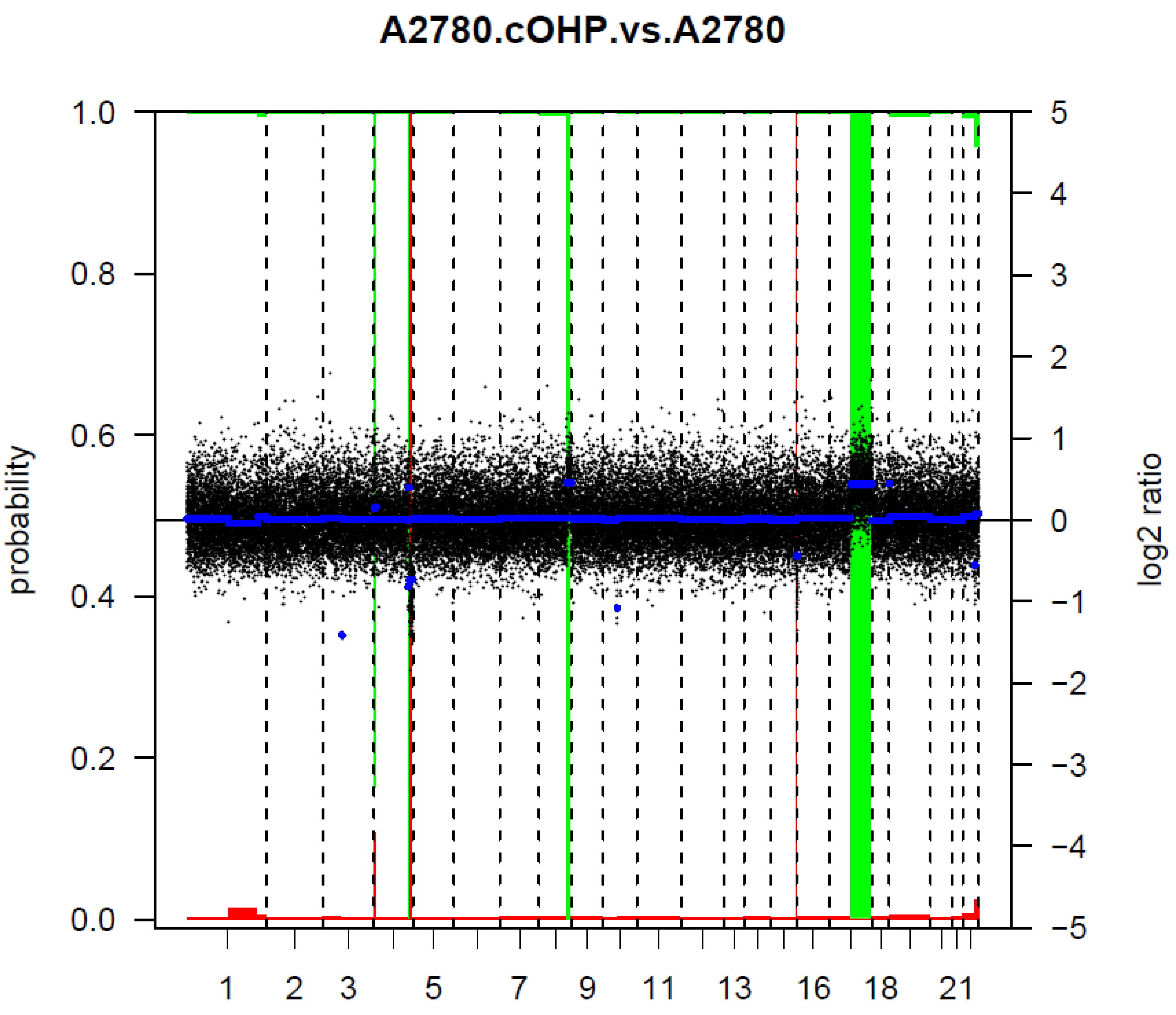
A2780.cOHP.vs.A2780
probability
log2 ratio
1.0
0.8
0.6
0.4
0.2
0.0
5
4
3
2
1
0
−1
−2
−3
−4
−5
1
2
3
5
7
9
11
13
16
18
21

ADDP.vs.A2780

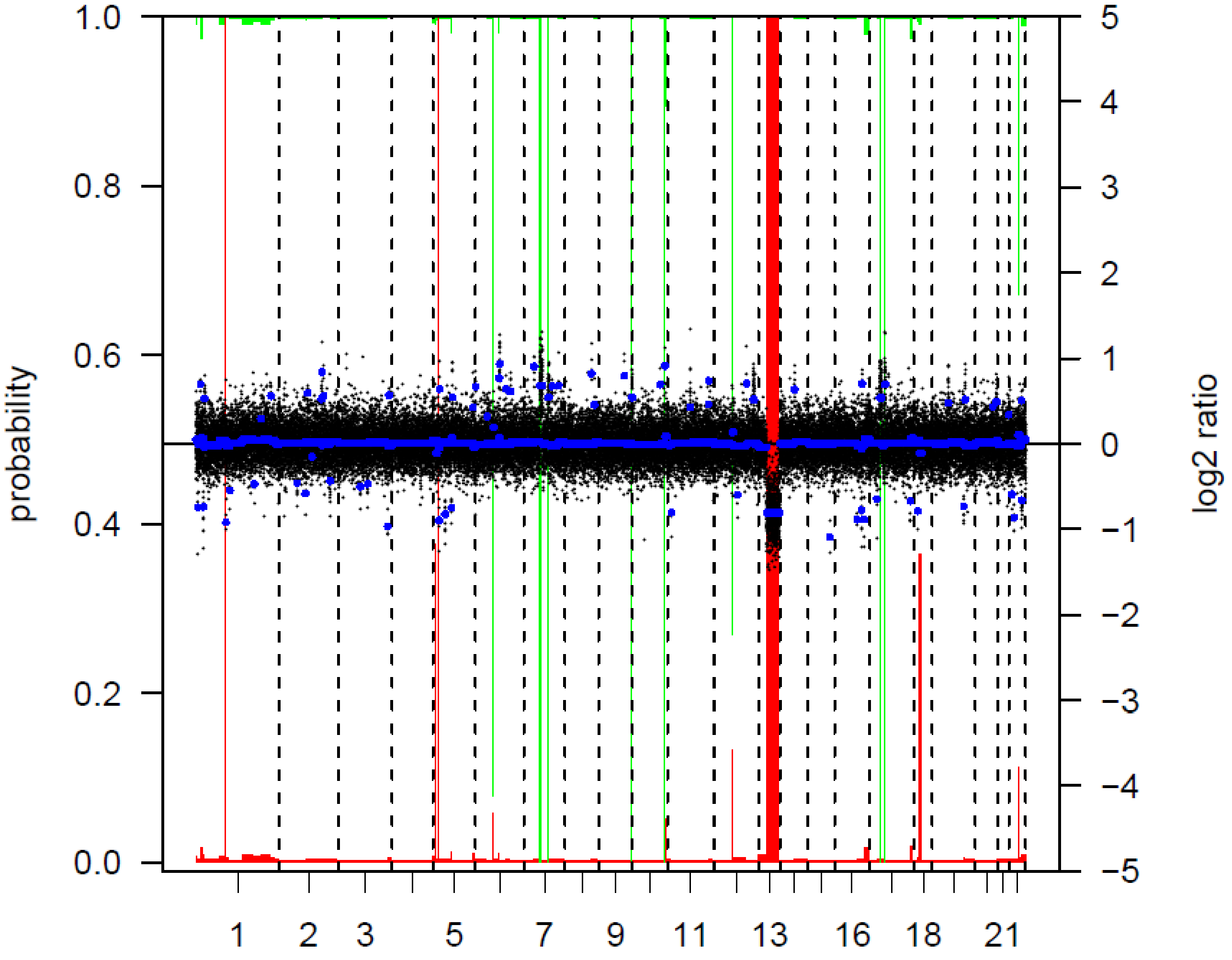

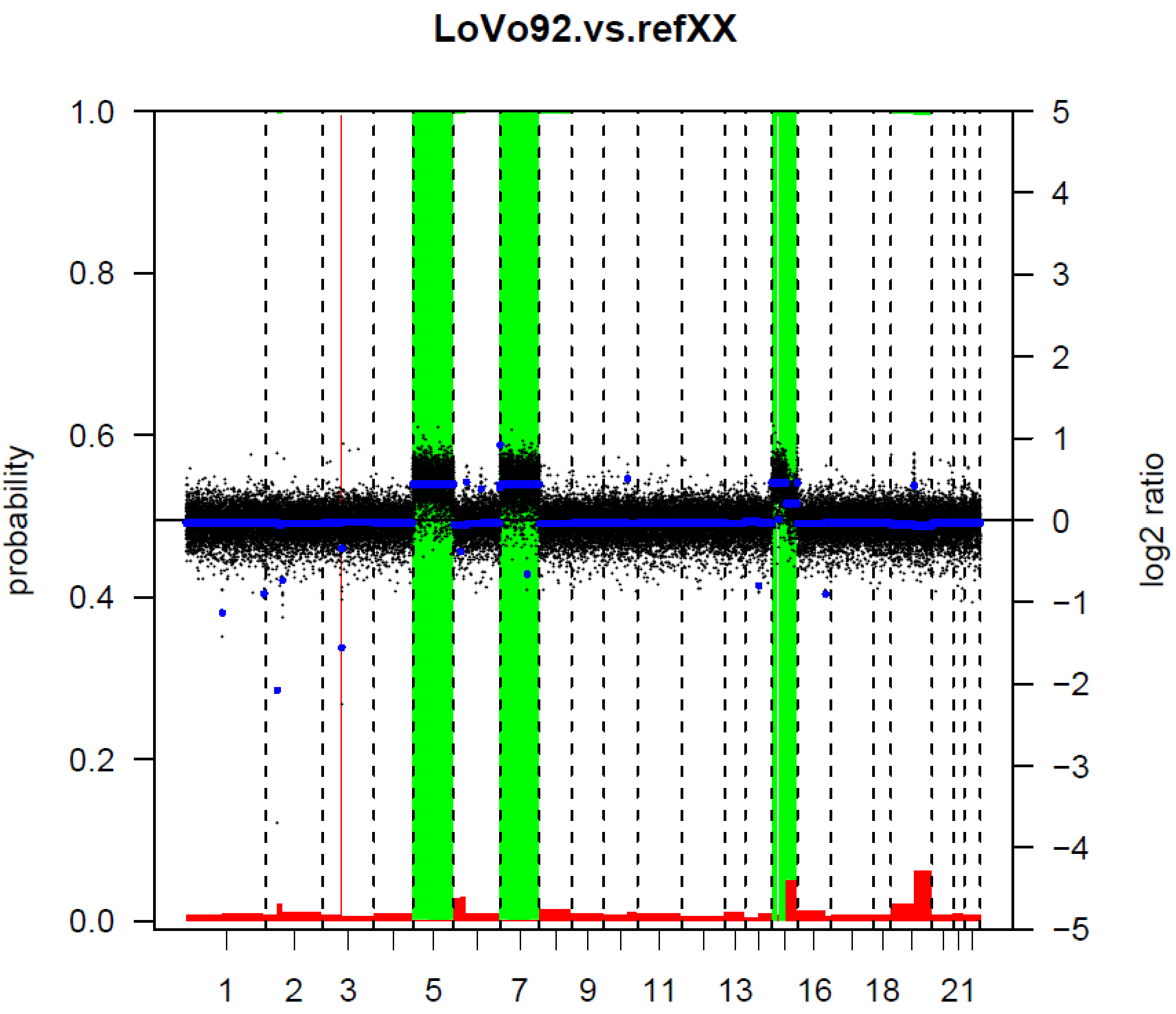
LoVo92.vs.refXX
probability
log2 ratio
1.0
0.8
0.6
0.4
0.2
0.0
5
4
3
2
1
0
−1
−2
−3
−4
−5
1
2
3
5
7
9
11
13
16
18
21

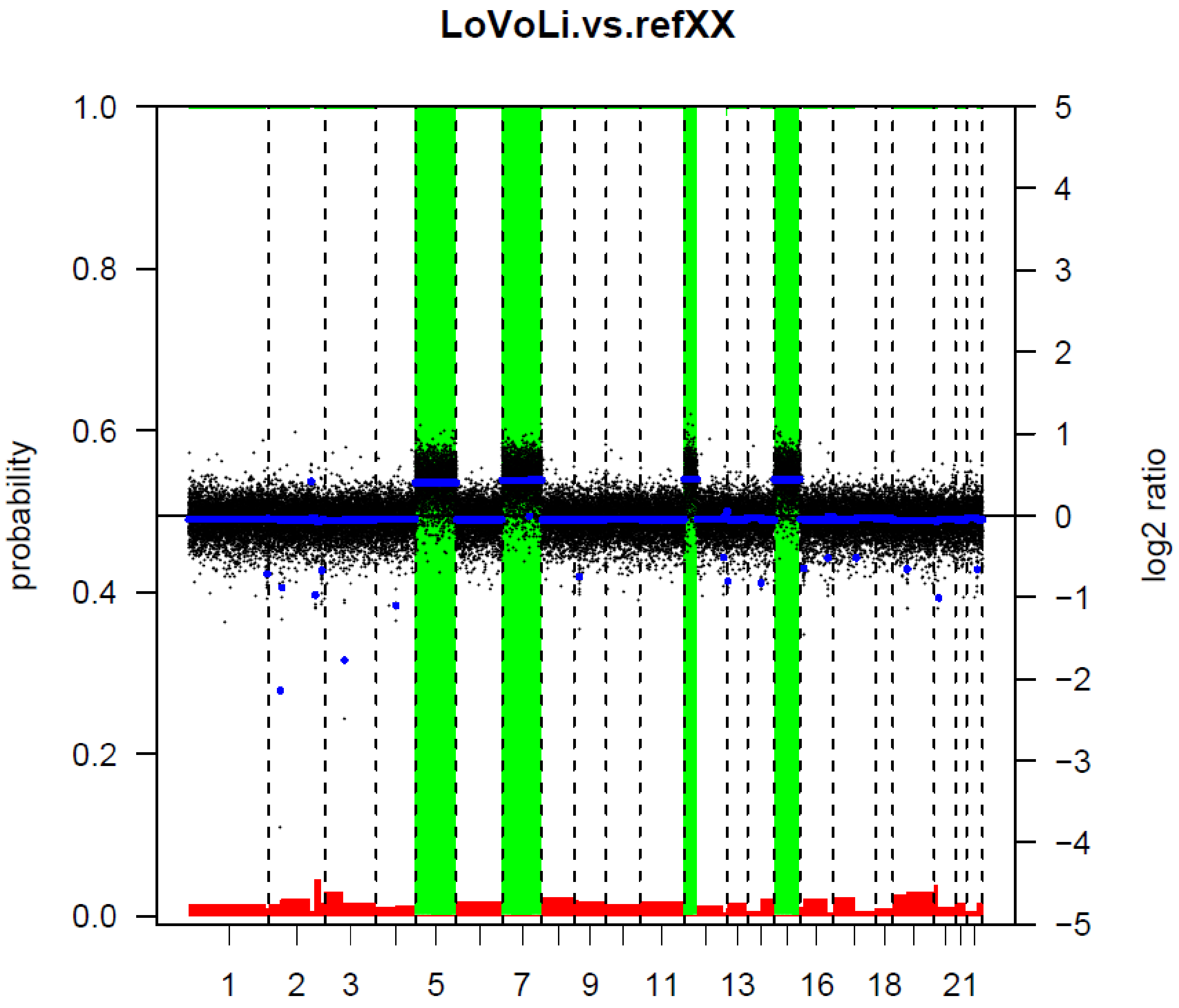
LoVoLi.vs.refXX
probability
log2 ratio
1.0
0.8
0.6
0.4
0.2
0.0
5
4
3
2
1
0
−1
−2
−3
−4
−5
1
2
3
5
7
9
11
13
16
18
21

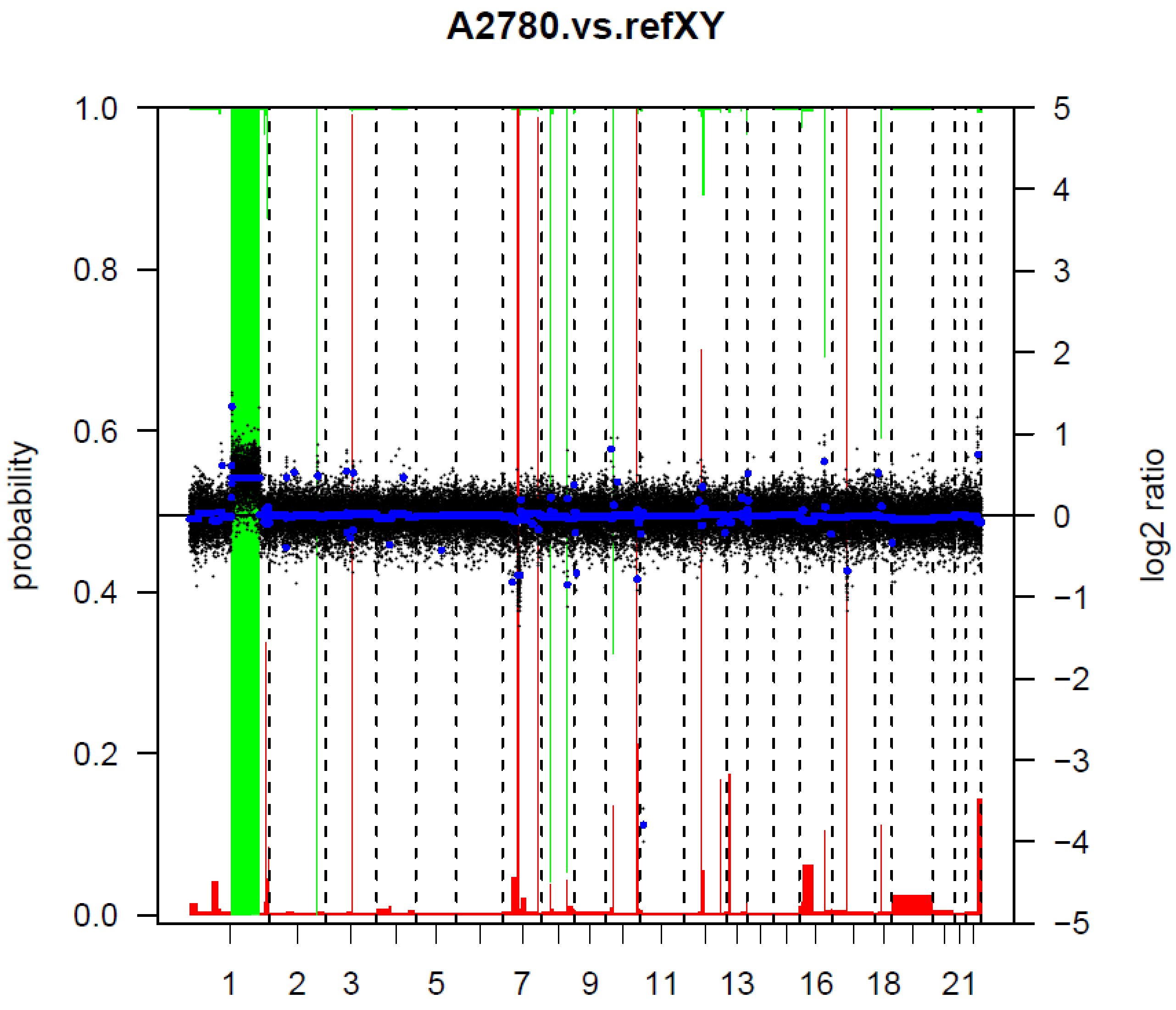

© 2019 by the authors. Licensee MDPI, Basel, Switzerland. This article is an open access article distributed under the terms and conditions of the Creative Commons Attribution (CC BY) license (http://creativecommons.org/licenses/by/4.0/).
